# The Vagus Nerve and Spleen: Influence on White Adipose Mass and Histology of Obese and Non-obese Rats

**DOI:** 10.3389/fphys.2021.672027

**Published:** 2021-06-25

**Authors:** Joice Cristina Kuchler, Bruna Schumaker Siqueira, Vanessa Marieli Ceglarek, Fernanda Vigilato Chasko, Isllany Carvalho Moura, Bruna Fatima Sczepanhak, Jean Franciesco Vettorazzi, Sandra Lucinei Balbo, Sabrina Grassiolli

**Affiliations:** ^1^Postgraduate Program in Applied Health Sciences, Western Paraná State University, Francisco Beltrão, Brazil; ^2^Laboratory of Endocrine and Metabolic Physiology, Postgraduate Program in Biosciences and Health, Western Paraná State University, Cascavel, Brazil; ^3^Department of Physiology, Institute of Basic Health Sciences, Federal University of Rio Grande do Sul, Porto Alegre, Brazil; ^4^Postgraduate Program in Biological Sciences, Physiology, Federal University of Rio Grande do Sul, Porto Alegre, Brazil; ^5^Educational Union of Cascavel, UNIVEL, Cascavel, Brazil

**Keywords:** vagotomy, splenectomy, autonomic nervous system, adipocyte, hypothalamic obesity

## Abstract

The vagus nerve (VN) and spleen represent a complex interface between neural and immunological functions, affecting both energy metabolism and white adipose tissue (WAT) content. Here, we evaluated whether vagal and splenic axis participates in WAT mass regulation in obese and non-obese male Wistar rats. High doses of monosodium glutamate (M; 4 g/Kg) were administered during the neonatal period to induce hypothalamic lesion and obesity (M-Obese rats). Non-obese or Control (CTL) rats received equimolar saline. At 60 days of life, M-Obese and CTL rats were randomly distributed into experimental subgroups according to the following surgical procedures: sham, subdiaphragmatic vagotomy (SV), splenectomy (SPL), and SV + SPL (*n* = 11 rats/group). At 150 days of life and after 12 h of fasting, rats were euthanized, blood was collected, and the plasma levels of glucose, triglycerides, cholesterol, insulin, and interleukin 10 (IL10) were analyzed. The visceral and subcutaneous WAT depots were excised, weighed, and histologically evaluated for number and size of adipocytes as well as IL10 protein expression. M-Obese rats showed higher adiposity, hyperinsulinemia, hypertriglyceridemia, and insulin resistance when compared with CTL groups (*p* < 0.05). In CTL and M-Obese rats, SV reduced body weight gain and triglycerides levels, diminishing adipocyte size without changes in IL10 expression in WAT (*p*< 0.05). The SV procedure resulted in high IL10 plasma levels in CTL rats, but not in the M-Obese group. The splenectomy prevented the SV anti-adiposity effects, as well as blocked the elevation of IL10 levels in plasma of CTL rats. In contrast, neither SV nor SPL surgeries modified the plasma levels of IL10 and IL10 protein expression in WAT from M-Obese rats. In conclusion, vagotomy promotes body weight and adiposity reduction, elevating IL10 plasma levels in non-obese animals, in a spleen-dependent manner. Under hypothalamic obesity conditions, VN ablation also reduces body weight gain and adiposity, improving insulin sensitivity without changes in IL10 protein expression in WAT or IL10 plasma levels, in a spleen-independent manner. Our findings indicate that the vagal-spleen axis influence the WAT mass in a health state, while this mechanism seems to be disturbed in hypothalamic obese animals.

## Introduction

White adipose tissue (WAT) exerts a central role in energy homeostasis, a function related to the endocrine activities of adipocytes (Ghaben and Scherer, 2019). Adipocytes present a narrow association between metabolism (lipogenesis and lipolysis), cell size (larger and small cells), and adipokine secretion (pro or anti-inflammatory substances) (Gustafson and Smith, 2015). Thus, in obesity conditions, the pronounced WAT expansion is primarily characterized by increased lipogenesis, adipocyte hypertrophy, and increases in pro-inflammatory proteins, such as tumor necrosis factor-alpha (TNFα) and interleukin 1 beta (IL1β), with simultaneous reduction in anti-inflammatory substances, such as interleukin 10 (IL10) and adiponectin (Van Meijel et al., 2019).

The imbalance between cellular and secretory functions of WAT is a key point for the development of metabolic abnormalities, such as insulin resistance, hyperglycemia, dyslipidemia, and hypertension, characterizing the metabolic syndrome (MS) (Klöting and Blüher, 2014; Chu et al., 2018). However, the origin of these processes is unknown. In this sense, the interplay of neuronal and immunological aspects seems to have an important impact in metabolic diseases, including those associated with WAT expansion (Seoane-Collazo et al., 2015). Two central arms in the immune and metabolic interface are the bi-directional influence of the vagus nerve (VN) and spleen on WAT function (Martin et al., 2015; Pavlov and Tracey, 2017; Ai et al., 2018).

Autonomic nervous system (ANS) imbalance is commonly observed in obesity, with VN hyperactivity involved in hyperinsulinemia, insulin resistance, glucose intolerance, and excessive WAT mass expansion (Cohen et al., 2013; Balbo et al., 2016). The WAT vagal innervation is a matter of discussion for several research groups (Kreier et al., 2002; Giordano et al., 2006). In this regard, some argue a lack of significant vagal innervation in WAT, while others report the presence of a parasympathetic input in WAT (Giordano et al., 2006; Holland et al., 2019). Independent of these discussion points, it is clear that VN ablation (vagotomy) induces WAT mass reduction, a response observed in obese human (Miyato et al., 2012) and rodent obesity models (Andrews et al., 1985; Balbo et al., 2016). The impact of VN ablation in adiposity probably involves neuro-immune system interplay with repercussions on metabolic state. In this sense, the reduction in brain-melanocortin signaling promotes fat mass gain, by activating the lipogenic program in adipocytes and the proliferation of endothelial cells in WAT depots, a response dependent of the efferent hepatic VN branch (Holland et al., 2019). Moreover, VN modulates WAT content by controlling the sympathetic peripheral tonus via central nervous system (CNS). Subdiaphragmatic vagotomy impairs the brown adipose tissue (BAT)-mediated diet-induced thermic response (Andrews et al., 1985), while acute VN stimulation increases norepinephrine concentrations and transmission in the rat brain (Follesa et al., 2007).

A well-known vagal-immune interaction is the anti-inflammatory vagal reflex (Pavlov and Tracey, 2017) for which the spleen is required (Rosas-Ballina et al., 2015). The immunological activities of the spleen are modulated by the ANS and the best recognized anti-inflammatory pathway in this organ is the sympathetic activity via the splanchnic nerve (Martelli et al., 2019). However, more recent data suggest that parasympathetic vagal activity is also able to alter immune splenic responses, being functionally relevant for the sympathetic tone control of the spleen (Rosas-Ballina et al., 2015). Despite the VN does not directly innervate splenic cells, the vagal preganglionic fibers synapse with postganglionic sympathetic neurons in celiac ganglion, subsequently traveling through splenic nerves (Berthoud and Neuhuber, 2019). As such, the sympathetic nervous system (SNS) and the VN synergically act through the splenic nerve, to inhibit the release of TNFα by macrophages in the spleen (Pavlov and Tracey, 2017).

Beside the known neuro-immune responses involving the spleen, this organ also affects energy homeostasis (John and King, 1914; Ai et al., 2018). Splenectomy changes WAT content, glucose, and lipids homeostasis, and insulin sensitivity in obese rodents (Leite et al., 2015). Obesity provokes fat accumulation and induces higher inflammatory responses in the spleen (Turbitt et al., 2019). Leptin, the primary WAT adipokine, increases SNS flux to the spleen when centrally administered (Tanida et al., 2019). Interestingly, the spleen is also an important source of the anti-inflammatory cytokine IL10, and several splenic metabolic functions could be a consequence of changes in IL10 secretion or action. For example, infusion of adipose tissue-derived stem cells (ADSCs) reduces hyperglycemia and insulin resistance in diabetic rats, a response dependent on spleen-derived IL10 expression (Zhang et al., 2017). Obesity is hypothesized to suppress the synthesis of IL10, resulting in chronic inflammation in WAT (Gotoh et al., 2012a). In obese humans, IL10 expression in WAT was inversely associated with insulin resistance (Mclaughlin et al., 2014). Interesting, adipocytes are able to modulate immune responses in the spleen, including IL10 production (Vielma et al., 2013; Toda et al., 2020). Moreover, WAT depots also represent an abundant source of IL10 in the context of viral infections (Garcia-Valtanen et al., 2020). IL10 knockout mice develop systemic inflammation and alterations in mitochondrial lipid metabolism (de-Lima-Júnior et al., 2019). Finally, VN stimulation increases IL10 endogenous production, a response mediated by splanchnic nerves in the spleen (Komegaea et al., 2018). Together, these data suggest a bi-directional vagal-splenic interaction impacting WAT mass through mechanisms still unknown.

Elevated doses of neonatal glutamate monosodium (M) administered to newborn rats induce hypothalamic lesions, primarily affecting the arcuate nucleus (ARC), resulting in massive adiposity associated with insulin resistance, glucose intolerance, and dyslipidemia, reproducing central elements of MS (Balbo et al., 2007; Grassiolli et al., 2007). Vagal hyperactivity, spleen abnormalities, and altered IL10 plasmatic levels have already been confirmed in M-Obese rats (Caetano et al., 2017; Guareschi et al., 2019). Based on these findings, M-Obese rats can be considered a substantial obesity model for investigating possible vagal–spleen interactions via IL10 actions and their repercussions on WAT histology and function. Thus, in the present work, we evaluated the effect of vagotomy associated with splenectomy on WAT content and histology in hypothalamic M-Obese and non-obese male Wistar rats, assessing whether changes in IL10 plasma levels or IL10 protein expression in WAT are involved in vagal-splenic responses.

## Materials and Methods

### Animals

Pregnant Wistar rats (*n* = 10) were obtained from the central animal facility of the Western Paraná State University (Unioeste) and transferred to the sectorial animal facility of the Laboratory of Endocrine and Metabolic Physiology (LAFEM). Animals were allocated into individual cages and received water and rodent chow (Biobase; SC; BR) *ad libitum* until the birth of offspring. At birth, the offspring size was adjusted to 6–8 male pups per dam, which were maintained under controlled luminosity cycles (12 h light–dark) and temperature (23 ± 2°C) during the lactation phase. All experimental procedures were approved by the local Ethics Committee on Animal Use (CEUA) on March 16, 2017, according to the Brazilian guidelines of the National Council for the Control of Animal Experimentation (CONCEA).

### Hypothalamic Obesity

On the second day after birth, the offspring were divided into two groups. One group (*n* = 44) received a daily subcutaneous injection of monosodium glutamate (MSG) in a dose of 4 g/Kg of body weight (bw) during five consecutive days according to a previously established protocol (Olney, 1969). Elevated MSG doses to neonates provoke hypothalamic lesions, inducing lifelong obesity (Timper and Brüning, 2017; Torrezan et al., 2019). This group was denominated M-Obese. Non-obese or Control rats (CTL; *n* = 44) received equimolar saline solution. After weaning (21 days of life), M-Obese and CTL animals were randomly distributed into cages (three rats/cage) and at 60 days of life subdivided according to the surgical procedures described below.

### Surgery Protocols

**FIGURE 1:**
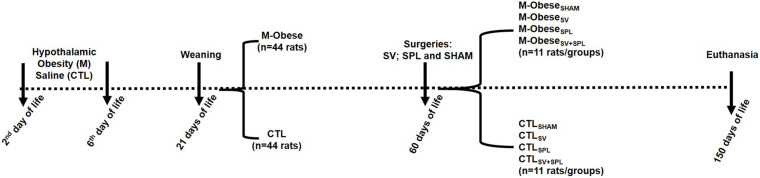
Experimental design. CTL animals were divided into CTL_SHAM_, Sham surgery control; CTL_SPL_, splenectomized control; CTL_SV_, subdiaphragmatic vagotomy control; CTL_SV__+__SPL_, subdiaphragmatic vagotomy + splenectomized control; M-Obese_SHAM_, simulated surgery MSG; M-Obese_SPL_, Splenectomized MSG; M-Obese_SV_, subdiaphragmatic vagotomy MSG; M-Obese_SV__+__SPL_, subdiaphragmatic vagotomy + splenectomized MSG *N* = number of animals. The dashed line indicates the events over time (from birth to euthanasia), with vertical arrows evidencing specific points.

At 60 days of life, M-Obese (*n* = 22) and CTL (*n* = 22) groups were submitted to subdiaphragmatic vagotomy (SV) and/or splenectomy (SPL) (Balbo et al., 2007; Gotoh et al., 2012b). Briefly, after 12 h of fasting, animals were anesthetized with isoflurane (1%) and maintained in spontaneous ventilation with oxygen (1 ml/min). Then, the abdominal cavity was opened throughout an incision (± 2 cm) performed immediately below the sternum. Liver was moved for visualization of the anterior and posterior VN ramus in the esophagus wall. The VN ramus were placed away from the esophageal wall, tied, and posteriorly sectioned. For splenic surgery, the abdominal incision was done as described above, the blood vessel connected to the spleen was tied, and the organ excised and weighed. A group of animals had both SV and SPL surgeries performed in the same procedure, while another group was submitted to a sham surgery. At the end, eight experimental groups were originated (*n* = 11 rats), as illustrated in Figure 1. After the surgical procedures, all rats were transferred to individual cages, receiving water and rodent chow *ad libitum* for 1 week, to guarantee postoperative recovery. After this period, animals were regrouped (three rats/cage) according to the surgical protocol until 150 days of life.

### Biometric and Plasma Parameters

From 70 to 150 days of life, rats were weighed for body weight gain (g) calculation. At 150 days of life, the naso-anal length (NAL; cm) was evaluated, and after 12 h of fasting, rats were euthanized, total blood was collected in heparinized tubes, and plasma used for dosage of glucose, triglycerides, and total cholesterol by enzymatic kits (Bioloquid, Laborclin, Pinhais, Brazil). Plasma insulin was measured by radioimmunoassay. Glucose and triglycerides values in fasting were used for TyG Index calculation using the formula: log [triglycerides (mg/dl)^∗^glucose (mg/dl)/2] (Guerrero-Romero et al., 2010). Plasma samples were also used for IL10 dosage by enzyme-linked immunosorbent assay (ELISA), according to the manufacturer’s instructions (Novex, Bender MedSystems GmbH, Vienna, Austria). Immediately after euthanasia, abdominal cavity was opened, the stomach was excised, emptied, cleaned, and the net weight registered. Stomach’s weight was used as a parameter of SV efficacy (Campfield et al., 1983). In non-splenectomized rats, the spleen was also excised, cleaned and the net weight registered. The final body weight and NAL were used to obtain the Lee index [^3^√body weight (g)/NAL (cm)]. The Lee index is a biomarker of adiposity in obese rodents, including those with hypothalamic obesity as previously proposed (Bernardis and Patterson, 1968).

### White Adipose Tissue Histological Analysis

After euthanasia, the mesenteric (WAT-M) and inguinal (WAT-I) WAT depots were excised, weighed, and a fragment was immediately transferred to Alfac, a histological fixation solution constituted by a mixture of alcohol (80%), formol (10%), and glacial acetic acid (5%), during 24 h. After this period, the WAT tissue samples were transferred to an alcoholic (70%) solution for histological procedures. For this, WAT depots were diaphanized in xylol, dehydrated in alcoholic solution and embedded in paraplast (McCormickTM; Leica Microsystems Pty Ltd., Sydney, Australia), being finally submitted to the microtomy procedures. Semi-serial cuts (5 μm) were performed and stained with hematoxylin and eosin (H&E). Tissues from five to six rats per group were used to assemble the slides for histology (three slides per rat, containing at leastthree slices each). Images of the slides were captured using a photomicroscope (Olympus BX60, Tokyo, Japan) at a magnification of 40x. Adipocytes, size (μm^2^), and number (number/field) were measured using an image analysis system (Image J 1.39f, NIH–Bethesda, MD, United States). A total of 50 adipocytes were analyzed per section.

### White Adipose Tissue Western Blotting

Fragments of WAT-M and WAT-I depots were homogenized in 200 μl of lysis buffer (10 mM EDTA, 100 mM Tris base, 100 mM sodium pyrophosphate, 100 mM sodium fluoride, 10 mM sodium orthovanadate, 2 mM phenylmethylsulfonyl fluoride, 1% triton X-100, and 1 μg/ml aprotinin). Protein concentration was measured using Bradford reagents (SIGMA, B6916). Of the protein samples, 30 μg was homogenized and boiled (5 min at 100°C) in Laemmli buffer. Proteins were then separated by electrophoresis in a 15% polyacrylamide gel. The transfer to nitrocellulose membranes was performed in Trans Blot transfer for 2 h at 110 V, with Tris/glycine buffer. Membranes were blocked in a Tris-buffered saline [10 mM tris base, 150 mM NaCl and 0.25% (vol./vol.) of tween 20] containing 5% (wt./vol.) of non-fat milk for 1 h at room temperature. After blocking, membranes were incubated overnight at 4°C with primary antibodies against IL10 (sc-8438–Santa Cruz, Dallas, Texas, United States) and Tubulin (sc-5286–Santa Cruz, Dallas, Texas, United States). Detection of specific protein bands was performed by incubating membranes with appropriate secondary antibodies (sc-2005–Santa Cruz, Dallas, Texas, United States) and bands detection was performed by measuring chemiluminescence (LOCCUS). Band’s intensity was quantified by optical densitometry using the software LabImageID. All other reagents were purchased from Sigma-Aldrich (St. Louis, MO, United States), unless otherwise stated.

### Statistical Analysis

Data are presented as mean ± standard error of the mean (SEM). CTL and M-Obese groups were compared using Student’s *t*-test (*p*< 0.05). The main outcome measurements were analyzed by two-way ANOVA, followed by Tukey’s *post hoc* test (*p*< 0.05). *Glass’s Delta*, for effect size (ES) evaluation, was also calculated and physiological relevance was interpreted as small (*d* = 0.2), medium (*d* = 0.5), or large (*d* = 0.8).

## Results

### White Adipose Tissue Visceral and Subcutaneous Hypertrophy, Dyslipidemia, and Insulin Resistance in M-Obese Rats

As shown in Table 1, hypothalamic lesions induced by MSG neonatal treatment had a large effect on biometric and biochemical parameters in adult life, as evidenced by the *ES*-values from M-Obese rats. Thus, at 150 days of life, M-Obese rats had lower body weight (29%; *ES* = −3.83), NAL (16%; *ES* = −10.30), and spleen weight (26%; *ES* = −1.29) compared with the CTL group (*p*< 0.001). In contrast, M-Obese rats showed higher Lee index (6%; *ES* = 2.59) compared with CTL animals, confirming elevated adiposity (*p*< 0.05). Moreover, the M-Obese group presented elevated plasma values of triglycerides (44%; *ES* = 2.44) and hyperinsulinemia (48%; *ES* = 1.60) compared with CTL rats (*p*< 0.05). The TyG index was approximately 6% higher in the M-Obese (*ES* = 2.01) group than in CTL animals. Although Delta’s glass analysis has pointed medium ES to fasting glucose (−0.78), IL10 plasma levels (0.68) and stomach weight (0.55) in M-Obese rats did not significantly differ (Student’s *t*-test, *p*> 0.05) from CTL animals (Table 1).

**TABLE 1:** Adiposity, biometric, and metabolic profile of M-Obese rats.

Parameters	CTL	M-Obese	*p*-value	ES
Body weight (g)	433 ± 9	306 ± 5	<0.0001	−3.83
NAL (cm)	23.26 ± 0.11	19.36 ± 0.28	<0.0001	−10.30
Lee Index	0.32 ± 0.02	0.34 ± 0.02	0.0008	2.59
WAT-I (g/100 g)	0.27 ± 0.03	0.55 ± 0.05	0.0007	3.12
WAT-M (g/100 g)	0.99 ± 0.060	2.26 ± 0.25	0.0004	7.89
Stomach (g/100 g)	0.48 ± 0.01	0.49 ± 0.02	0.6064	0.55
Spleen (g/100 g)	0.14 ± 0.01	0.10 ± 0.01	0.0049	−1.29
Insulinemia (ng/ml)	0.09 ± 0.02	0.18 ± 0.02	0.0206	1.60
Glucose (mg/dl)	93 ± 8	75 ± 6	0.1124	−0.78
Triglycerides (mg/dl)	121.1 ± 16	217.1 ± 35	0.0337	2.44
TyG	2.00 ± 0.03	2.13 ± 0.04	0.0157	2.01
Cholesterol (mg/dl)	113 ± 3.8	146 ± 16.4	0.0722	3.25
IL-10 (pg/ml)	39.31 ± 4.57	50.13 ± 3.94	0.1122	0.68

*Data are expressed as mean ± SEM, n = 8–11 rats per group.*

*NAL, naso-anal length; TyG, triglycerides and glucose index; IL10, interleukin 10; g, grams; cm, centimeters; mg, milligrams; dl, deciliters; ng, nanograms; ml, milliliters; CTL, control; M-Obese, MSG; ES, Effect size; WAT-I, inguinal white adipose tissue; WAT-M, mesenteric white adipose tissue; Student’s t-test (p < 0.05).*

**FIGURE 2:**
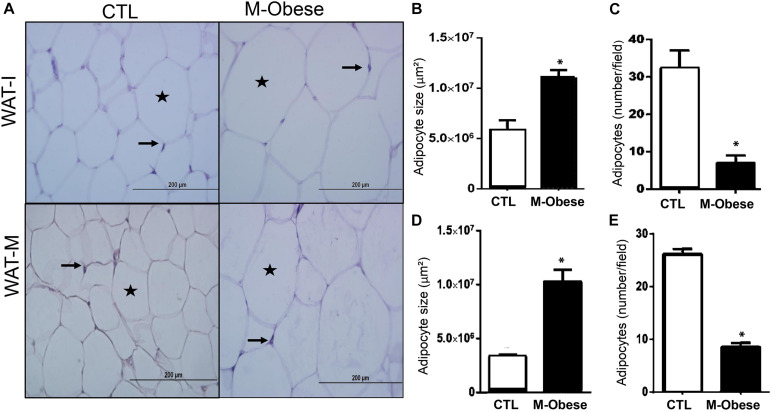
M-Obese rats show intense adipocyte hypertrophy in WAT. **(A)** Representative photomicrographs of WAT-I and WAT-M, respectively, stained with H&E, under light microscopy, 40-fold magnification; **(B,D)** illustrate the average adipocyte size; **(C,E)** number of adipocytes per field in WAT-I and WAT-M, respectively. Adipocyte nuclei are indicated by arrows, and the deposition of fat in the cytosol is marked by the star. CTL, control; M-Obese, MSG; WAT-I, inguinal white adipose tissue; WAT-M, mesenteric white adipose tissue. Data are mean ± SEM (*n* = 6 rats/group). Asterisk (^∗^) represent statistical differences between the groups. Student’s *t*-test (*p* < 0.05).

High fat content was observed in WAT-M (128%; *ES* = 7.89) and WAT-I (103%; *ES* = 3.12) of M-Obese rats compared with the CTL group (Table 1; *p*< 0.0001). M-Obese rats displayed adipocyte hypertrophy in WAT-I (86%; *ES* = 2.19) and WAT-M (209%; *ES* = 17.67) depots, where we consequently observed a reduction in the number of adipocytes (WAT-I: 72%; *ES* = −5.96 and WAT-M: 68%; *ES* = −7.36) in comparison with adipocytes from CTL group (Figures 2A–D; *p* < 0.0001). Additional representative photomicrographs of WAT-I and WAT-M depots in the CTL and M-Obese groups are showed in Supplementary Figures 1a–d.

### Vagus Nerve Ablation Reduces Body Weight Gain and Adiposity in Non-obese Rats (CTL) in a Spleen-Influenced Manner

As a consequence of the SV surgery, the stomach weight was 53% and 52% higher in CTL_SV_ and CTL_SV__+__SPL_ groups, respectively, in comparison with CTL_SHAM_ animals (*p*< 0.0001; Table 2). The SV surgery affected body weight gain [*F*_(__1_._40__)_ = 0.0823; *p* = 0.0001] and NAL [*F*_(__1_._30__)_ = 11.10; *p*< 0.0023]. Thus, the body weight gain was reduced by 34.2% and 34.6% in CTL_SV_ and CTL_SV__+__SPL_, respectively, compared with CTL_SHAM_ group (*p*< 0.05). Similar results were also observed when vagotomized rats were compared with the CTL_SPL_ group (Table 2). Moreover, the CTL_SV_ and CTL_SV__+__SPL_ rats also displayed significant reductions in NAL when compared with non-vagotomized groups (CTL_SHAM_ and CTL_SPL_; *p*< 0.05). However, the Lee index was not affected by SV surgery. In contrast, spleen ablation did not affect body weight gain or NAL, but influenced the Lee index [*F*_(__1_._28__)_ = 5.033; *p* = 0.0330]. The spleen weight was similar between CTL_SHAM_ and CTL_SV_ groups (Table 2; *p* < 0.05).

**TABLE 2:** Effects of subdiaphragmatic vagotomy (SV) and/or splenectomy (SPL) surgeries on biometric and biochemical parameters from non-obese rats (CTL).

						*p*-value	
	CTL_SHAM_	CTL_SPL_	CTL_SV_	CTL_SV+SPL_	SPL	SV	I
Body weight gain (g)	149 ± 5.1^c,d^	129 ± 4.8^c,d^	98 ± 7.4^a,b^	98 ± 10^a,b^	0.2290	0.0001	0.2098
NAL (cm)	23.16 ± 0.14^c^	23.00 ± 0.26	22.12 ± 0.28^a^	22.44 ± 0.25	0.7521	0.0023	0.3186
Lee index	0.32 ± 0.002	0.31 ± 0.004	0.32 ± 0.003	0.31 ± 0.002	0.0330	0.7143	0.8095
Stomach (g/100 g)	0.48 ± 0.01^c,d^	0.52 ± 0.03^c,d^	1.04 ± 0.06^a,b^	1.02 ± 0.05^a,b^	0.8762	< 0.0001	0.4618
Spleen (g/100 g)	0.14 ± 0.01	N/A	0.13 ± 0.01	N/A		0.2828	
Glucose (mg/dl)	93 ± 8^d^	74 ± 5^d^	103 ± 11^d^	152 ± 17^a,b,d^	0.2055	<0.0010	0.0075
Insulinemia (ng/ml)	0.09 ± 0.02	0.10 ± 0.03	0.19 ± 0.03	0.19 ± 0.02	0.9126	0.0049	0.8911
Cholesterol (mg/dl)	113 ± 3.8	202 ± 35.2	147 ± 23	179 ± 28	0.0304	0.8226	0.2928
Triglycerides (mg/dl)	120 ± 13	129 ± 10^d^	88 ± 5.7	78 ± 14^b^	0.9433	0.0018	0.4220
TyG	2.03 ± 0.03	1.97 ± 0.02	1.96 ± 0.03	2.05 ± 0.02	0.6856	0.9318	0.0102
IL10 (pg/ml)	30.17 ± 3.29^b^	36.17 ± 4.04^a,d^	56.43 ± 10.72	25.11 ± 2.18^b^	0.0271	0.1537	0.0027

*Data are expressed as mean ± SEM (n = 8–11 rats/group). ANOVA two-way F-values are shown in SPL, SV, and I column. Letters above numbers indicate statistical difference among groups—(a) CTL_*SHAM*_; (b) CTL_*SP*_; (c) CTL_*SV*_; (d) CTL_*SV*__+__*SPL*_ in Tukey post hoc test (p < 0.05).*

*SPL, splenectomy; V, subdiaphragmatic vagotomy; I, Interaction; NAL, naso-anal length; TyG, Triglycerides and glucose index; IL-10, Interleukin 10; g, grams; cm, centimeters; mg, milligrams; dl, deciliters; ng, nanograms; ml, milliliters; N/A, not applicable. CTL_*SHAM*_, surgical simulation control; CTL_*SPL*_, splenectomized control; CTL_*SV*_, subdiaphragmatic vagotomy control; CTL_*SV*__+__*SPL*_, subdiaphragmatic vagotomy + splenectomized control; WAT-I, white adipose tissue–inguinal; IL-10, Interleukin 10; SPL, splenectomy; SV, subdiaphragmatic vagotomy; I, interaction.*

SV surgery modified the fasting glycemia [*F*_(__1_._31__)_ = 13.33; *p* = 0.0010] with interaction between SV surgery and spleen ablation in this variable [*F*_(__1_._31__)_ = 81.96; *p* = 0.075]. Thus, CTL_SV__+__SPL_ rats showed hyperglycemia when compared with other experimental groups (Table 2; *p*< 0.001). Insulin and total cholesterol during fasting were influenced by SV [*F*_(__1_._24__)_ = 9.616; *p* = 0.0049] and SPL [*F*_(__1_._27__)_ = 5.22; *p* = 0.0304] surgeries, respectively. However, we did not observe significant difference in Tukey’s post-test (Table 2; *p*> 0.05). In contrast, SV surgery modified triglycerides plasma levels [*F*_(__1_._24__)_ = 12.32; *p* = 0.0018], which were reduced in CTL_SV__+__SPL_ animals in comparison with CTL_SPL_ rats (*p*< 0.05). Although the combination of SV and SPL surgeries [*F*_(__1_._24__)_ = 7.783; *p* = 0.0102] influenced TyG values, as shown in Table 2, no statistical difference between the groups was identified by Tukey’s post-test. Plasma levels of IL10 were influenced by SPL factor [*F*_(__1_._16__)_ = 5.922; *p* = 0.0271], as well as by the interaction between SPL and SV surgeries [*F*_(__1_._16__)_ = 12.56; *p* = 0.0027]. CTL_SV__+__SPL_ rats showed lower IL10 plasma levels in comparison with CTL_SV_ group, while CTL_SV_ animals presented higher IL10 plasma levels when compared with other experimental groups (*p* < 0.05).

**FIGURE 3:**
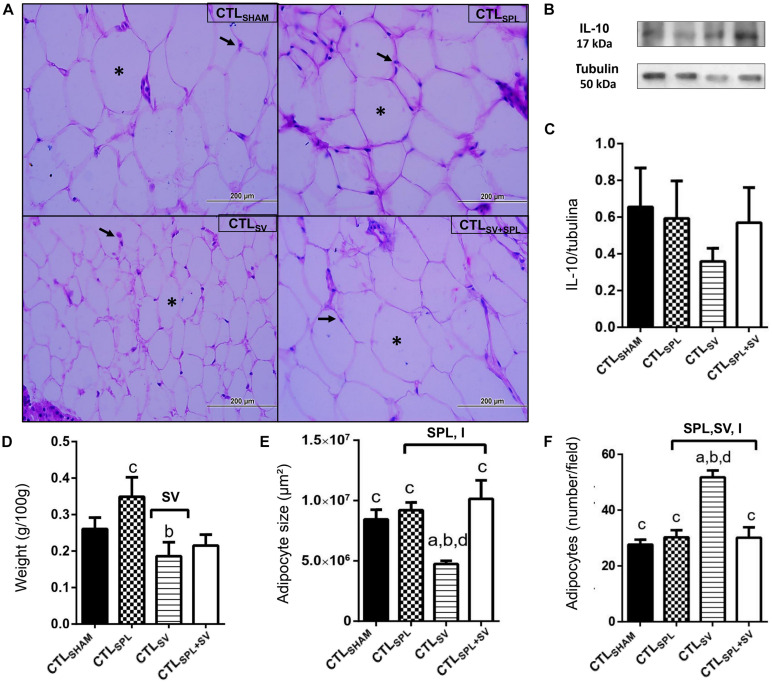
Subdiaphragmatic vagotomy induces a reduction in WAT-I content in non-obese (CTL) rats with splenic participation and without changes in IL10 protein expression. **(A)** Representative photomicrographs of the broad WAT-I, stained with H&E, magnification 40×; adipocyte nuclei (arrow) and fat deposition (asterisk). **(B)** Representative WB band densitometry; 50 kDa region (tubulin); 17 kDa region IL10; In graphical, data are mean ± SEM **(C)** IL-10 expression (*n* = 4 rats/group); **(D)** weight of WAT-I (*n* = 6 rats/group); **(E)** size of adipocytes; **(F)** number of adipocytes (*n* = 6 rats/group). Line and symbols (SPL, SV, and I) above bars show significant F effect for two-way ANOVA. Letters represent statistical difference among groups–(a) CTL_SHAM_; (b) CTL_SPL_; (c) CTL_SV_; (d) CTL_SV__+__SPL_ in Tukey *post hoc* test (*p* < 0.05). Legend: CTL_SHAM_, simulated surgery control; CTL_SPL_, splenectomized control; CTL_SV_, subdiaphragmatic vagotomy control; CTL_SV__+__SPL_, subdiaphragmatic vagotomy + splenectomized control; WAT-I, white adipose tissue–inguinal; IL-10, Interleukin 10; SPL, splenectomy; SV, subdiaphragmatic vagotomy; I, interaction.

**FIGURE 4:**
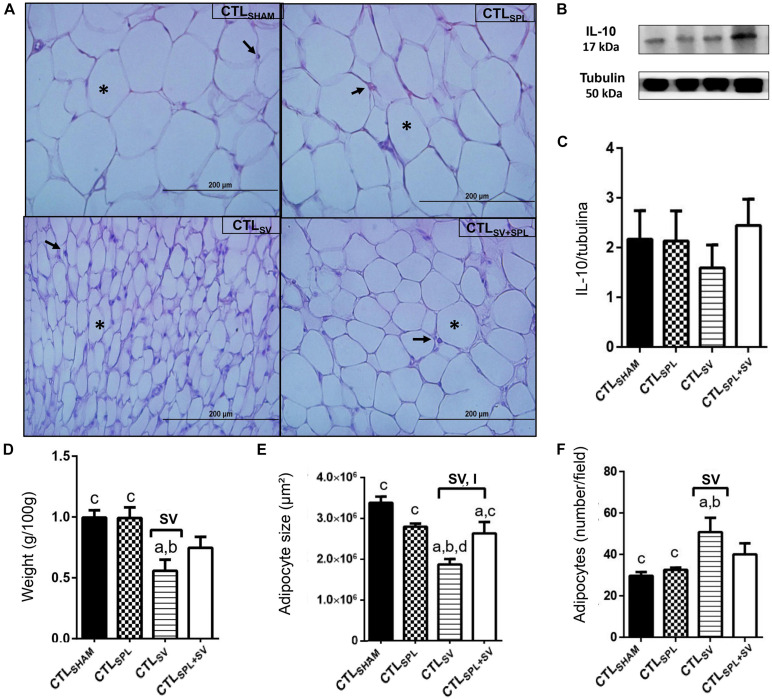
Subdiaphragmatic vagotomy induces reduction in WAT-M content in non-obese (CTL) rats with splenic participation and without changes IL10 protein expression. **(A)** Representative photomicrographs of the broad WAT-M, stained with H&E, magnification 40×; adipocyte nuclei (arrow) and fat deposition (asterisk). **(B)** Representative WB band densitometry; 50 kDa region (tubulin); 17 kDa region IL10; In graphical, data are mean ± SEM **(C)** IL-10 expression (*n* = 4 rats/group); **(D)** weight of WAT-I (*n* = 6 rats/group); **(E)** size of adipocytes; **(F)** number of adipocytes (*n* = 6 rats/group). Line and symbols (SPL, SV, and I) above bars show significant F effect in Two-way ANOVA. Letters represent statistical difference among groups—(a) CTL_SHAM_; (b) CTL_SPL_; (c) CTL_SV_; (d) CTL_SV__+__SPL_ in Tukey *post hoc* test (*p* < 0.05). Legend: CTL_SHAM_, simulated surgery control; CTL_SPL_, splenectomized control; CTL_SV_, subdiaphragmatic vagotomy control; CTL_SV__+__SPL_, subdiaphragmatic vagotomy + splenectomized control; WAT-I, white adipose tissue—inguinal; IL-10, Interleukin 10; SPL, splenectomy; SV, subdiaphragmatic vagotomy; I, interaction.

The impact of SV and SPL surgeries on adiposity content and histology from non-obese rodents (CTL) are presented in Figures 3A–F, 4A–F. The adiposity content was significantly influenced by SV surgery in both WAT-I [*F*_(__1_._29__)_ = 7.163; *p* = 0.0121] and WAT-M [*F*_(__1_._28__)_ = 16.50; *p* = 0.0004] depots. Thus, CTL_SV_ animals presented lower WAT-I weight in comparison with CTL_SPL_ rats (Figure 3D; *p*< 0.05). In addition, in WAT-I depots from the CTL_SV_ group, we observed a reduction in adipocyte size (Figure 3E; *p*< 0.05) and a consequent increase in adipocytes number (Figure 3F; *p*< 0.05) in relation to other groups, as shown in the representative photomicrography (Figure 3A). The weight of WAT-M in CTL_SV_ group was also reduced by 35 and 40%, respectively, in relation to CTL_SHAM_ and CTL_SPL_ rats (*p*< 0.05; Figure 4A). Thus, in WAT-M depots from CTL_SV_ rats, adipocyte size was reduced (Figure 4E; *p*< 0.05), and adipocytes numbers were consequently increased (Figure 4F; *p*< 0.05) in comparison with CTL_SHAM_ animals, as shown in the representative photomicrography (Figure 4A).

Spleen ablation alone did not alter the content of WAT-I and WAT-M depots when compared with CTL_SHAM_ groups (Figures 3D, 4D); *p*> 0.05). However, CTL_SPL_ rats were found to have higher WAT-I and WAT-M mass weight in relation to CTL_SV_ animals (Figure 3D; *p*< 0.05). Moreover, interaction between SV-SPL surgeries influenced size [*F*_(__1_._65__)_ = 6.613; *p* = 0.0124; Figure 3E] and number [*F*_(__1_._65__)_ = 20.63; *p* = 0.0001 Figure 3F] of adipocytes in WAT-I, and also affected WAT-M adipocytes size [*F*_(__1_._20__)_ = 14.54; *p* = 0.0011]. Thus, CTL_SV__+__SPL_ animals show that size and number of adipocytes in WAT-I and WAT-M depots are higher than CTL_SV_ rats and similar to CTL_SHAM_ rats (Figures 3E,F, 4E,F). Additional representative photomicrographs of WAT-I and WAT-M depots from CTL rats submitted to SV or SPL surgeries rats are showed in Supplementary Figures 1e–l). Both SV and SPL surgeries did not modify IL10 expression neither in WAT-I (Figures 3B,C) nor in WAT-M depots (Figures 4B,C) in CTL groups.

### Vagus Nerve Ablation Induces Preferential White Adipose Tissue Visceral Content Reduction in M-Obese Rats, Improving Insulin Sensitivity Without Changes in IL10 Plasma Levels or Expression in White Adipose Tissue

The VN ablation induced significant increases in stomach weight in M-Obese_SV_ and M-Obese_SV__+__SPL_ rats in relation to M-Obese_SHAM_ groups, confirming gastric stasis (Table 3; *p* = 0.0001). In the M-Obese groups, SV [*F*_(__1_._27__)_ = 69.31; *p*< 0.0001] and SV + SPL combination [*F*_(__1_._27__)_ = 4.461; *p* = 0.0441] affected stomach weight. M-Obese_SV_ and M-Obese_SPL_ groups showed significant reduction in approximately 24% in body weight gain in comparison with M-Obese_SHAM_ rats (*p*< 0.05). Moreover, M-Obese_SV_ rats had lower body weight gain in relation to M-Obese_SPL_ animals (*p*< 0.05). Neither SV nor SPL surgeries altered NAL and spleen weight in M-Obese animals. However, SV surgery affected the Lee index [*F*_(__1_._26__)_ = 7.933; *p* = 0.0091] resulting in smaller Lee index value in M-Obese_SV__+__SPL_ rats compared with M-Obese_SHAM_ animals (Table 3; *p*< 0.05). Fasting values of glucose and total cholesterol were not modified by SV and/or SPL surgeries in M-Obese groups (*p*> 0.05). In contrast, SPL surgery affected fasting insulin plasma levels [*F*_(__1_._23__)_ = 7.862; *p* = 0.0101] resulting in lower levels in M-Obese_SV_ animals when compared with the M-Obese_SV__+__SP__L_ groups (Table 3; *p*< 0.05). Moreover, SV surgery affected plasma triglycerides levels [*F*_(__1_._17__)_ = 6.573; *p* = 0.0201], but without a difference in Tukey’s post-test between M-Obese groups (Table 3; *p*> 0.05). Thus, the TyG index was influenced by SV surgery [*F*_(__1_._15__)_ = 25.67; *p* = 0.0001] in M-Obese groups. Therefore, we found smaller values of TyG index in M-Obese_SV_ and M-Obese_SV__+__SPL_ groups in relation to M-Obese_SHAM_ rats (Table 3; *p*< 0.05). M-Obese rats did not show significant changes in IL10 plasma levels in any of the surgical procedures.

**TABLE 3:** Effect of SV and/or SPL surgeries on biometric and biochemical parameters of M-Obese animals.

						*p*-value	
	M-Obese_SHAM_	M-Obese_SPL_	M-Obese_SV_	M-Obese_SV+SPL_	SPL	SV	I
Weight gain (g)	132 ± 9^c,d^	117 ± 3^c^	88 ± 5^a,b^	100 ± 5^a^	0.7855	0.0001	0.0322
NAL (cm)	19.3 ± 0.28	19.8 ± 0.3	20.1 ± 0.4	19.8 ± 0.4	0.8143	0.3359	0.3096
Lee index	0.343 ± 0.003^d^	0.334 ± 0.003	0.329 ± 0.005	0.327 ± 0.003^a^	0.1047	0.0091	0.3600
Stomach (g/100 g)	0.49 ± 0.02^c,d^	0.45 ± 0.01^c,d^	0.85 ± 0.06^a,b^	1.04 ± 0.10^a,b^	0.2008	<0.0001	0.0441
Spleen (g/100 g)	0.102 ± 0.002	N/A	0.100 ± 0.003	N/A		0.6974	
Glucose (mg/dl)	75 ± 6	81 ± 5	85 ± 6	89 ± 2	0.3650	0.0961	0.8604
Insulinemia (ng/ml)	0.18 ± 0.02	0.21 ± 0.01	0.13 ± 0.02^d^	0.23 ± 0.02^c^	0.0101	0.4976	0.1309
Cholesterol (mg/dl)	146 ± 16	131 ± 33	116 ± 26	126 ± 32	0.9420	0.6015	0.7203
Triglycerides (mg/dl)	217 ± 31	236 ± 32	145 ± 27	150 ± 23	0.6945	0.0201	0.8149
TyG	2.16 ± 0.03^c,d^	2.10 ± 0.02	2.00 ± 0.02^a^	2.00 ± 0.02^a^	0.2878	0.0002	0.2017
IL10 (pg/ml)	53.58 ± 5.85	57.56 ± 7.62	61.53 ± 8.67	61.37 ± 14.95	0.5635	0.5042	0.8985

*Data are expressed as mean ± SEM (n = 8–11 rats/group). ANOVA two-way, F-values are shown in SPL, SV, and I columns. Letters above numbers indicate statistical difference among groups–(a) M-Obese_*SHAM*_; (b) M-Obese_*SPL*_; (c) M-Obese_*SV*_; (d) M_–_Obese_*SV*__+__*SPL*_ in Tukey post hoc test (p < 0.05).*

*SPL, splenectomy; SV, subdiaphragmatic vagotomy; I, interaction; NAL, nasoanal length; TyG, triglycerides and glucose index; IL-10, interleukin 10; g, grams; cm, centimeters; mg, milligrams; dl, deciliters; ng, nanograms; ml, milliliters; N/A, not applicable; M-Obese_*SHAM*_, surgical simulation, MSG; M-Obese_*SPL*_, splenectomized MSG; M-Obese_*SV*_, subdiaphragmatic vagotomy MSG; M-Obese_*SV*__+__*SPL*_, subdiaphragmatic vagotomy + splenectomized MSG.*

**FIGURE 5:**
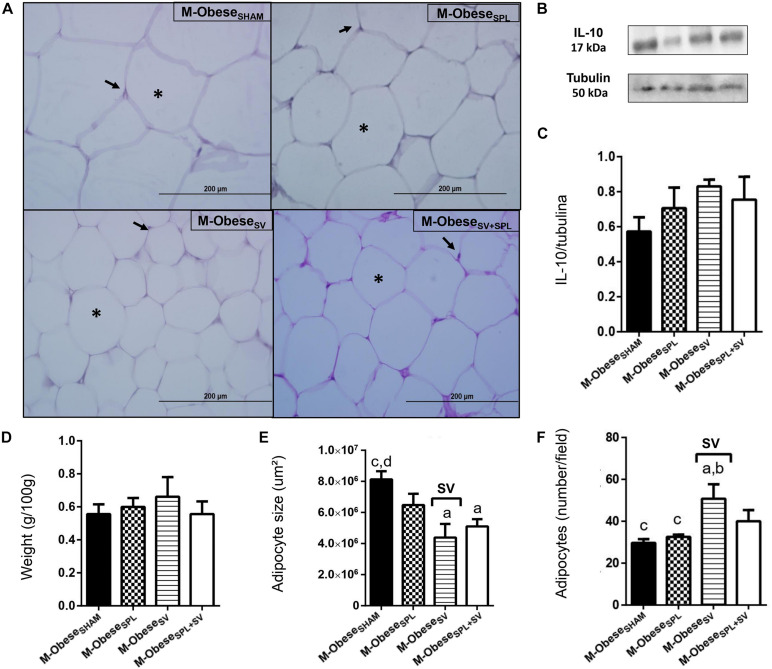
Subdiaphragmatic vagotomy does not change WAT-I content in M-Obese rats but reduces adipocytes hypertrophy, without altering IL10 expression, regardless of spleen ablation. **(A)** Representative photomicrographs of the broad WAT-I, stained with H&E, magnification 40×; adipocyte nuclei (arrow) and fat deposition (asterisk). **(B)** Representative WB band densitometry; 50 kDa region (tubulin); 17 kDa region IL10; In graphical, data are mean ± SEM **(C)** IL-10 expression (*n* = 4 rats/group); **(D)** weight of WAT-I (*n* = 6 rats/group); **(E)** size of adipocytes; **(F)** number of adipocytes (*n* = 6 rats/group). Line and symbols (SPL, SV, and I) above bars show significant F effect in Two-way ANOVA. Letters represent statistical difference among groups—(a) M-Obese_SHAM_; (b) M-Obese_SPL_; (c) M-Obese_SV_; (d) Obese_SV__+__SPL_ in Tukey *post hoc* test (*p* < 0.05). Legend: M-Obese_SHAM_, simulated surgery MSG; M-Obese_SPL_, splenectomized MSG; M-Obese_SV_, subdiaphragmatic vagotomy MSG; M-Obese_SV__+__SPL_, subdiaphragmatic vagotomy + splenectomized MSG; WAT-I, white adipose tissue–inguinal; IL10, Interleukin 10; SPL, splenectomy; SV, subdiaphragmatic vagotomy, I, interaction.

**FIGURE 6:**
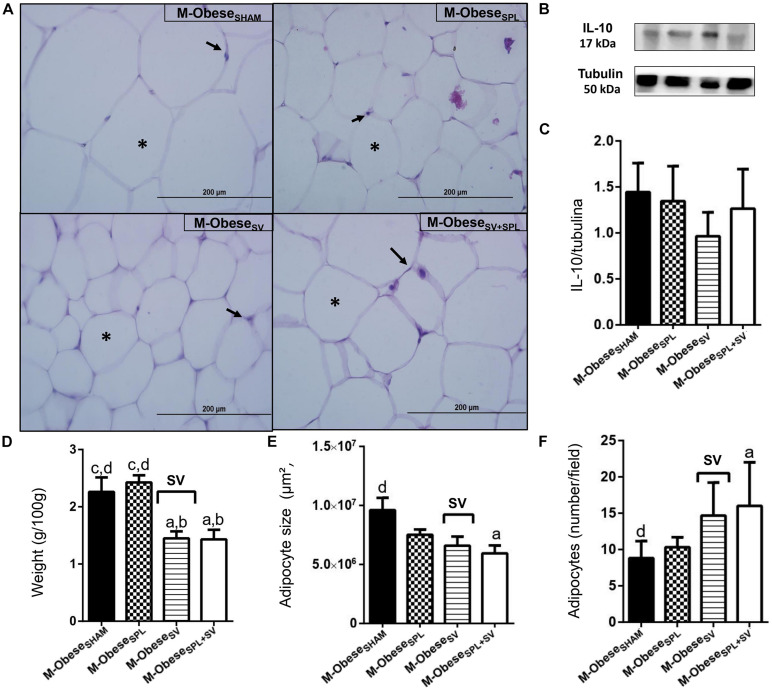
Subdiaphragmatic vagotomy induces a reduction in WAT-M content in M-Obese rats, which is enhanced in the absence of the spleen, without leading to changes in IL10 expression. **(A)** Representative photomicrographs of the broad WAT-M, stained with H&E, magnification 40×; adipocyte nuclei (arrow) and fat deposition (asterisk). **(B)** Representative WB band densitometry; 50 kDa region (tubulin); 17 kDa region IL10; In graphical, data are mean ± SEM **(C)** IL-10 expression (*n* = 4 rats/group); **(D)** weight of WAT-I (*n* = 6 rats/group); **(E)** size of adipocytes; **(F)** number of adipocytes (*n* = 6 rats/group). Line and symbols (SPL, SV, and I) above bars show significant F effect in two-way ANOVA. Letters representing statistical difference among groups—(a) M-Obese_SHAM_; (b) M-Obese_SPL_; (c) M-Obese_SV_; (d) Obese_SV__+__SPL_ in Tukey *post hoc* test (*p* < 0.05). Legend: M-Obese_SHAM_, simulated surgery MSG; M-Obese_SPL_, splenectomized MSG; M-Obese_SV_, subdiaphragmatic vagotomy MSG; M-Obese_SV__+__SPL_, subdiaphragmatic vagotomy + splenectomized MSG; WAT-I, white adipose tissue–inguinal; IL-10, Interleukin 10; SPL, splenectomy; SV, subdiaphragmatic vagotomy; I, interaction.

The influence of SV and/or SPL surgeries on adiposity content and histology of M-Obese rats are shown in Figures 5A–F; WAT-I) and Figures 6A–F; WAT-M). The content of WAT-I was not affected by SV or SPL in M-Obese rats (Figure 5D; *p*> 0.05). However, the adipocytes size [*F*_(__1_._16__)_ = 13.83; *p* = 0.0019] and number [*F*_(__1_._17__)_ = 16.25; *P* = 0.0009] were modified by SV factor in M-Obese animals. Adipocyte size was significantly smaller in M-Obese_SV_ and M-Obese_SV__+__SPL_ groups in relation to M-Obese_SHAM_ rats (Figures 5E,F; *p*< 0.05). The number of adipocytes was elevated in WAT-I from M-Obese_SV_ group in comparison to M-Obese_SHAM_ animals (Figure 5F; *p*< 0.05). The WAT-M content was influenced by SV surgery in M-Obese rats [*F*_(__1_._29__)_ = 29.84; *p*< 0.0001], resulting in lower weight of WAT-M in M-Obese_SV_ rats when compared with M-Obese_SHAM_ and M-Obese_SPL_ groups (Figure 6D; *p*< 0.05). Moreover, adipocytes size [*F*_(__1_._20__)_ = 9.012; *p* = 0.0070] and number [*F*_(__1_._20__)_ = 12.42; *p* = 0.0021] in WAT-M depots were modified by SV factor, since adipocytes size was smaller, while adipocytes numbers were increased in M-Obese_SV__+__SPL_ rats in comparison with M-Obese_SHAM_ group (Figure 6F; *p*< 0.05). Representative photomicrography shows the effects of the surgical procedures in WAT-I (Figure 5A) and WAT-M (Figure 6A). Additional representative photomicrographs of WAT-I and WAT-M depots from M-Obese rats submitted to SV or SPL surgeries rats are showed in Supplementary Figures 1m–t. In contrast, IL10 expression was not modified by SV or SPL ablation in WAT-I (Figure 5B; *p*> 0.05) and WAT-M (Figure 6B; *p*> 0.05) depots.

### Effect Size of Subdiaphragmatic Vagotomy and Splenectomy Surgeries Presented Different Impact in Non-obese and M-Obese Rats

*Glass’s delta* analysis is a measure that enabled us to calculate the effect size (ES) of SV and/or SPL surgeries on CTL and M-Obese rats, considering their respective SHAM groups as internal controls. A schematic summary of ES is shown in Table 4, while ES values are presented in Supplementary Table 1. In CTL rats, VN ablation led to more pronounced effects, such as a reduction in body weight gain, anti-adiposity actions and a reduction in triglycerides plasma levels and TyG index, indicated by large negative ES values in SV groups. In contrast, CTL_SV_ groups presented elevated fasting insulin and IL10 plasma levels, since these variables show large positive *ES*-values (Supplementary Table 1). Excluding large positive *ES*-values for cholesterol plasma levels, SPL surgery showed, in general, a minor impact in the CTL group, with smaller or medium *ES*-values for other variables (Supplementary Table 1). Importantly, in CTL rats (non-obese), the spleen ablation changes the impact of vagotomy in adiposity and metabolic variables. For example, the positive small *ES*-value of SV in Lee index appears as large negative ES in the CTL_SV__+__SPL_ group. Similarly, the large negative ES of SV in TyG, WAT-I adiposity and IL10 plasma levels disappear in CTL_SV__+__SPL_ groups. On the other hand, glucose and cholesterol plasma levels were elevated by SV, and these effects were accented by splenectomy, having large *ES*-values in CTL_SV__+__SPL_ (Table 4 and Supplementary Table 1).

**TABLE 4:** *Glass’s delta* effect size in CTL (non-obese) and M-Obese rats.

	CTL (non-obese)			M-Obese		
	SHAM X SPL	SHAM X SV	SHAM X SPL + SV	SHAM X SPL	SHAM X SV	SHAM X SPL + SV
**Biometric**	**ES**	**ES**	**ES**	**ES**	**ES**	**ES**
BW gain	**↓ Large**	**↓Large**	**↓Large**	↓Medium	**↓Large**	**↓Large**
NAL	↓Small	**↓Large**	**↓Large**	↑Medium	**↑Large**	↑Medium
Lee index	**↓Large**	↑Small	**↓Large**	**↓Large**	**↓Large**	**↓Large**
Stomach w.	**↑Large**	**↑Large**	**↑Large**	↓Medium	**↑Large**	**↑Large**
Spleen w.	N/A	↓Small	N/A	N/A	↓Small	N/A
**Plasma**						
Glucose	**↓Large**	↑Small	**↑Large**	↑Small	↑Medium	↑Medium
Triglycerides	↑Small	**↓Large**	**↓Large**	↑Medium	**↓Large**	**↓Large**
TyG	↓Medium	**↓Large**	↔	**↓Large**	**↓Large**	**↓Large**
Insulin	↔	**↑Large**	**↑Large**	↑Small	**↓Large**	↑Medium
Cholesterol	**↑Large**	**↑Large**	**↑Large**	↓Small	↓Medium	↓Small
IL10	↑Medium	**↑Large**	↓Medium	↑Small	↑Medium	↑Medium
**WAT-I**						
Weight	**↑Large**	**↓Large**	↓Medium	↑Small	↑Medium	↔
Cell size	↑Small	**↓Large**	↑Medium	**↓Large**	**↓Large**	**↓Large**
Cell number	↑Small	**↑Large**	↑Small	↓Medium	**↑Large**	**↑Large**
IL10 protein	↔	↓Medium	↓Small	**↑Large**	**↑Large**	**↑Large**
**WAT-M**						
Weight	↔	**↓Large**	**↓Large**	↑Small	**↓Large**	**↓Large**
Cell size	**↓Large**	**↓Large**	**↓Large**	**↓Large**	**↓Large**	**↓Large**
Cell number	↑Medium	**↑Large**	**↑Large**	↑Medium	**↑Large**	**↑Large**
IL10 protein	↔	↓Medium	↑Small	↔	↓Medium	↓Small

*Glass’s delta effect size (ES) = small (d = 0.2–0.49), medium (0.5–0.79), and large (≥ 0.8). ES numeric values are shown in Supplementary Table 1. Legend: ↑ rise, ↔ no effect and ↓ reduction; Bold: Large ES; BW, body weight; NAL, naso-anal length; w, weight; WAT, white adipose tissue inguinal; WAT-M, white adipose tissue mesenteric; IL10, interleukin 10.*

In M-Obese rats a reduction in body weight gain and adiposity was also noted in vagotomized rats, with large negative *ES*-values (Table 4 and Supplementary Table 1). However, in contrast to the CTL group, in M-Obese rats, the SV increased NAL and reduced Lee index, resulting in larger negative *ES*-values. Importantly, in M-Obese groups, SV surgery modified insulin and triglycerides plasma levels, since these variables presented large negative ES, with repercussions in TyG index (Table 4 and Supplementary Table 1). The anti-adiposity impact of SV surgery was also noted in M-Obese groups with several *ES*-values in WAT-M and WAT-I depots. Thus, SV surgery in M-Obese rats provoked greater reduction in WAT-M content (larger negative *ES*-values; Supplementary Table 1) in comparison with WAT-I content. In both WAT depots from M-Obese rats were noted larger negative *ES*-values in size of adipocytes and a consequent large positive *ES*-values in numbers of adipocytes (Table 4 and Supplementary Table 1). In contrast, spleen ablation did not modify this effect of SV in M-Obese groups. Moreover, spleen absence in M-Obese rats also reduced adipocytes size in WAT, with larger negative *ES*-values (Table 4 and Supplementary Table 1).

## Discussion

It is widely accepted that ANS and immunological functions are differently modulated in obese and non-obese states and that changes in neuro-immune axis explain many comorbidities related to WAT mass expansion (Balbo et al., 2016; Mauer et al., 2016; Gotoh et al., 2017). Herein, we confirmed that SV surgery exerts anti-adiposity actions, promoting a reduction in body weight gain, WAT content, and adipocyte size, especially in non-obesity conditions. Importantly, the impact of VN ablation in adiposity in non-obese animals may be dependent on the presence of spleen and changes in IL10 plasma levels. On the other hand, in M-Obese rats, the response to VN ablation involves the restoration of insulin sensitivity, primarily reducing WAT visceral adipocyte hypertrophy and content.

Initially confirming previous studies by us (Grassiolli et al., 2007) and (Leite et al., 2015), we showed that neonatal administration of MSG promoted massive adiposity, insulin resistance, hypertriglyceridemia, and hyperinsulinemia. Moreover, excessive expansion of WAT in M-Obese rats was characterized by adipocyte hypertrophy in both visceral and subcutaneous WAT depots. Autonomic unbalance, with higher vagal hyperactivity and lower sympathetic tonus, is an evident phenomena in M-Obese rodents, which contributes to elevated WAT mass (Torrezan et al., 2019). In addition, in this obese rodent model, several hormonal and metabolic abnormalities contribute to elevated WAT mass and adipocyte hypertrophy, such as greater insulin lipogenic action (Kulyte et al., 2017), reduced lipolytic responses, higher cortisol and reduced growth hormone (GH) levels. Herein, we also confirmed the reduction of spleen weight in M-Obese rats, which may be related with histological alterations in white and red pulp in this organ, as previously demonstrated by our research group (Guareschi et al., 2019). Despite the spleen atrophy observed in the present study, we did not observe significant reductions in IL10 plasma levels in M-Obese rats, in contrast to a previous study (Caetano et al., 2017).

Our data corroborate previously published studies, showing that SV surgery reduces body weight gain and lowers adiposity in CTL and M-Obese animals (Balbo et al., 2007; Dezfuli et al., 2018), an effect that may relate to changes in food intake. The impact of VN ablation on food intake presents contradictory results, and time and technique surgery procedure are important aspects to consider when interpreting these results (Inoue and Bray, 1977; Louis-Sylvestre, 1983; Andrews et al., 1985). However, SV surgery did not alter food intake in non-obese or M-Obese animals (no published data). In this regard, SV causes a reduction in stomach motility and consequent gastric stasis, with higher food accumulation in this organ (Louis-Sylvestre, 1983; Andrews et al., 1985). Herein, the stomach weight from vagotomized rats (M-Obese and CTL) was significantly higher in relation to respective SHAM groups, indicating SV surgery efficacy.

Vagotomized CTL rats showed significant reduction in growth, suggesting an impact of VN in GH action or secretion in the non-obese state. Interestingly, ghrelin is a GH-releasing factor, which is altered by gastric stasis (Date, 2012). Our findings support a study (Al-Massadi et al., 2011) demonstrating that vagotomized animals downregulate GHRH mRNA in the ARC and downregulate mRNA of both GHRH and GHS receptors at the pituitary level, which are essential for the full GH-releasing effect of ghrelin. These responses were not observed in M-Obese animals probably due to extensive ARC lesions and a well-recognized reduction in GH release in M-Obese treated rats (Olney, 1969).

The VN plays an important role in glucose homeostasis, in particular, by modulating insulin secretion by the pancreas (Balbo et al., 2007), a response altered in obesity. In the present work we noted that fasting metabolic parameters were differently modulated by SV and SPL surgery in non-obese (CTL) and M-Obese animals. Thus, fasting glucose elevation was a consequence of VN ablation in CTL rats—an effect which was accentuated by spleen ablation—suggesting the participation of both the VN and the spleen in glucose homeostasis in the healthy state. Supporting this hypothesis, cervical VN stimulation (VNS) causes a rise in fasting glucose, reducing glucose tolerance in lean rats (Stauss et al., 2018), while hyperglycemia was more frequently observed in splenectomized humans (Ley et al., 2012).

In the present study, SV surgery reduced fasting insulin and improved insulin sensitivity in M-Obese rats, but not in CTL animals. As mentioned above, vagal hyperactivity is associated with hyperinsulinemia and insulin resistance in M-Obese rodents (Grassiolli et al., 2007; Balbo et al., 2007). The VN also exerts an impact in lipid metabolism, and VNS causes a reduction in plasma triglycerides in rats (Chen et al., 2018). According to our data, SV surgery also reduced triglyceride plasma levels in non-obese and M-Obese animals. However, it is important to note that the vagotomy-induced reduction in triglycerides was more pronounced (by *ES*-values) in M-Obese rats, confirming that vagal dysfunction has a role in dyslipidemia in this obese model (Lubaczeuski et al., 2015). In addition, vagotomy in M-Obese rats restored insulin sensitivity. Moreover, we have previously demonstrated that spleen ablation at 60 days of life did not alter glucose tolerance or insulin levels in CTL animals, but significantly reduces insulin levels and improves insulin sensitivity in M-Obese rats (Souza et al., 2020). Thus, ARC lesions in M-Obese rats possibly lead to VN hyperactivity and splenic dysfunction (Cohen et al., 2013) and therefore, the ablation of the VN and the spleen exerts a positive impact on glucose and lipid metabolism in this obese model.

The reduction in body weight gain and triglyceride levels observed in vagotomized, CTL and M-Obese rats may be partially explained by the lower adiposity found in these groups. However, the anti-adiposity effects of SV on WAT were more evident in CTL animals, in which we observed a greater reduction in adipocyte size in both visceral and subcutaneous WAT depots. These data suggested that, in the healthy state, the VN has a greater impact in fat mass regulation. Insulin is a central hormone for adipocyte lipogenesis and proliferation (Gustafson et al., 2015). Considering that in CTL rats, neither insulin levels nor insulin sensibility were affected by SV surgery, we believe that VN ablation in this case favors SNS lipolytic action in WAT. The VN appears to be able to affect peripheral SNS flux by a modulatory action on the nucleus of the solitary tract (NTS) and hypothalamic nucleus (Bonaz, 2020). In this regard, auricular VNS elevates norepinephrine levels in WAT depots (Chen et al., 2018). Moreover, as demonstrated by another study (Holland et al., 2019), the VN exerts effect on lipogenic pathways in WAT via melanocortin system responses at the hypothalamic level, suggesting that increased VN activity may have a role in the gain of fat mass.

In M-Obese animals, insulin fasting and insulin sensibility were improved by VN ablation, explaining the adipocyte size reduction. Thus, it is likely that SV surgery in M-Obese rats corrects vagal hyperactivity, restoring insulin action and exerting anti-adiposity effects, as suggested in other studies (Balbo et al., 2007). Similarly, clinical studies have demonstrated that surgical ablation of the abdominal VN can result in considerable reduction of body weight and vagal denervation has also been linked to increased weight loss following gastrectomy (Miyato et al., 2012). In the present work, we performed a total SV surgery, making it impossible to distinguish afferent from efferent vagal signals. However, in obesity, vagal afferent signals are also disturbed, suggesting that vagal blocking therapy can provide significant weight loss in obese patients (Ikramuddin et al., 2014; Apovian et al., 2017). Moreover, VNS is able to increase brown adipose tissue thermogenesis and promote brightening in WAT depot of obese rodents, favoring elevated energy expenditure and fat reduction (Van Meijel et al., 2019). Importantly, afferent vagal signals are conducted to the ARC nucleus (Miller, 2017), which is damaged in M-Obese rats. Thus, the interruption of peripheral vagal hyperactivity in M-Obese is a central anti-adiposity effect of SV in this obese model. Herein, we also noted that, in M-Obese animals, the visceral WAT depot was more responsive to VN ablation, presenting higher reduction in content in comparison to WAT-I. Corroborating this finding, selective VN denervation in obese animals submitted to gastrectomy resulted in preferential reduction of visceral WAT, indicating that VN locally regulates the amount of intra-abdominal fat tissue (Miyato et al., 2012).

The neuro-immunological axis is related with ANS innervation to immune organs, such as the spleen. Interestingly, the VN and spleen are involved in common responses, especially anti-inflammatory activities (DiSpirito and Mathis, 2015; Ai et al., 2018; Serhan and Levy, 2018). However, we Souza et al. (2020) and Wu et al. (2014) and Rosas-Ballina et al. (2015) have previously demonstrated that the spleen can also participate in glucose homeostasis and fat mass distribution. The results shown in the present work indicate, for the first time, that vagal–splenic signals could be participating in adiposity control, particularly in non-obese condition. Thus, we demonstrated that spleen ablation avoided an SV-induced reduction in WAT mass and adipocytes size suggesting that the vagal effects on WAT could be dependent on splenic activity in health state.

The spleen has been reported as an important site of IL10 production (Gotoh et al., 2017) and splenectomized rats showed reduction in IL10 plasma levels, associated with a pro-inflammatory effect on WAT. Moreover, splanchnic nerve stimulation regulates IL10-related splenic anti-inflammatory responses (Bonaz et al., 2016) via the beta adrenergic receptor; a VN-mediated process. In our study, however, splenectomy did not promote any significant reduction in IL10 plasma levels neither in CTL nor in M-Obese rats, suggesting that other sites may be contributing to IL10 plasma concentrations. Although the spleen is essential for anti-inflammatory reflex, other abdominal organs, such as the adrenal gland, may be involved in this response (Martelli et al., 2019). Interestingly, we observed that in CTL vagotomized animals, there was an increase in IL10 plasma levels in the presence, but not in the absence of a spleen. Thus, we speculated that in vagotomized CTL animals there is an augmented SNS firing rate to spleen, stimulating IL10 production. This hypothesis is supported by another study that found higher levels of norepinephrine in the spleen after vagotomy (Pongratz et al., 2012).

In addition, we also demonstrated that increased plasma levels of IL10 observed in vagotomized CTL animals does not appear to be dependent on WAT, since the expression of IL10 protein in visceral or subcutaneous WAT were not influenced by SV or SPL. The role of IL10 in WAT is largely unknown. Some studies have suggested that IL10 might create an anti-inflammatory milieu by promoting the activity of M2 macrophages (Lumeng et al., 2007; Almeida et al., 2019; Steen et al., 2020). In contrast, IL10 adipogenic and pro-inflammatory effects have also been reported (Acosta et al., 2019).

In our study, neither SV nor SPL ablation promoted significant alterations in IL10 plasma levels or IL10 expression in WAT from M-Obese rats, suggesting that the vagal-splenic circuits are interrupted in obesity. Similarly, other studies have not supported an anti-obesity role for IL-10 (Pongratz et al., 2012; Bonaz et al., 2016). In this sense, loss of IL10 expression in mice increased energy expenditure and protected against diet-induced obesity (Rajbhandari et al., 2018). We have previously demonstrated that the spleen of M-Obese rats display altered histological distribution of white splenic pulp (Guareschi et al., 2019), suggesting splenic dysfunction in this obesity model. However, to date, IL10 production in the spleen of M-Obese rats has not been studied. In addition, the participation of other cytokines in adipocyte vagal-splenic responses cannot be discharged. For example, the IL17 response by spleen cells has been demonstrated to be dependent on the presence of adipocyte-derived mediators (Silvana et al., 2016), suggesting that multiple cytokines participate in cross-directional interactions between spleen and adipocytes. Importantly, VN-splenic axis also modulates plasma levels of pro-inflammatory cytokines, such as, IL6 and TNFα, which are altered in this M-Obese rodent model, but have not been assessed in the present study.

In summary, we demonstrated that VN ablation has anti-adiposity effects on obese and non-obese rats. However, in non-obese animals, anti-adiposity effects of vagotomy on WAT are dependent on increased IL10 plasma levels and the presence of the spleen, suggesting that the vagal-splenic axis modulates the metabolism in health state. In contrast, in M-Obese animals, VN ablation restores insulin sensitivity and consequently reduces WAT visceral mass, without the participation of the spleen or IL10, pointing out a disrupted vagal-splenic axis in hypothalamic obesity.

## Data Availability Statement

The original contributions presented in the study are included in the article/Supplementary Material, further inquiries can be directed to the corresponding author.

## Ethics Statement

The animal study was reviewed and approved by Comit de tica no Uso de Animais (CEUA) da Universidade Estadual do Oeste do Paran.

## Author Contributions

JK: data analysis and interpretation, design of the work, critical revision of the article, and final approval of the version to be published. BSS: data collection, conception, design of the work, and final approval of the version to be published. VC: data analysis and interpretation, critical revision of the article, and final approval of the version to be published. FC, IM, and BFS: data collection, histological technique, and final approval of the version to be published. JV: molecular technique, critical revision of the article, and final approval of the version to be published. SB: surgical technique, critical revision of the article, and final approval of the version to be published. SG: conception, drafting the article, data analysis and interpretation, critical revision of the article, and final approval of the version to be published.

## Conflict of Interest

The authors declare that the research was conducted in the absence of any commercial or financial relationships that could be construed as a potential conflict of interest.
