# Supplementary material for: The Vagus Nerve and Spleen: Influence on White Adipose Mass and Histology of Obese and Non-obese Rats
Source: Front Physiol. 2021 Jun 25;12:672027. doi: 10.3389/fphys.2021.672027 (PMC8269450; doi:10.3389/fphys.2021.672027)
Supplement: Supplementary file 1 [file Image_1.pdf]

Figure S1. Representative photomicrographs of WAT-I and WAT-M from CTL and M-obese rats submitted to SV and/or SPL surgeries.

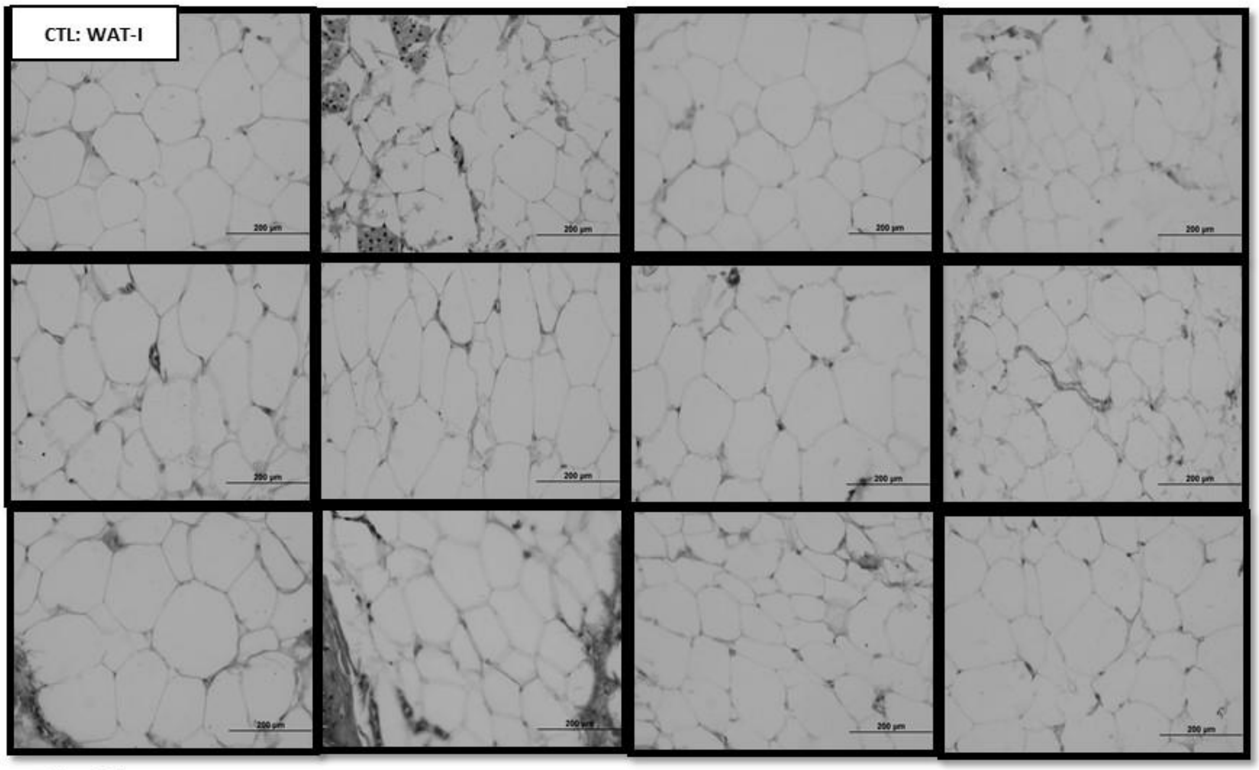

Fig. S1 a

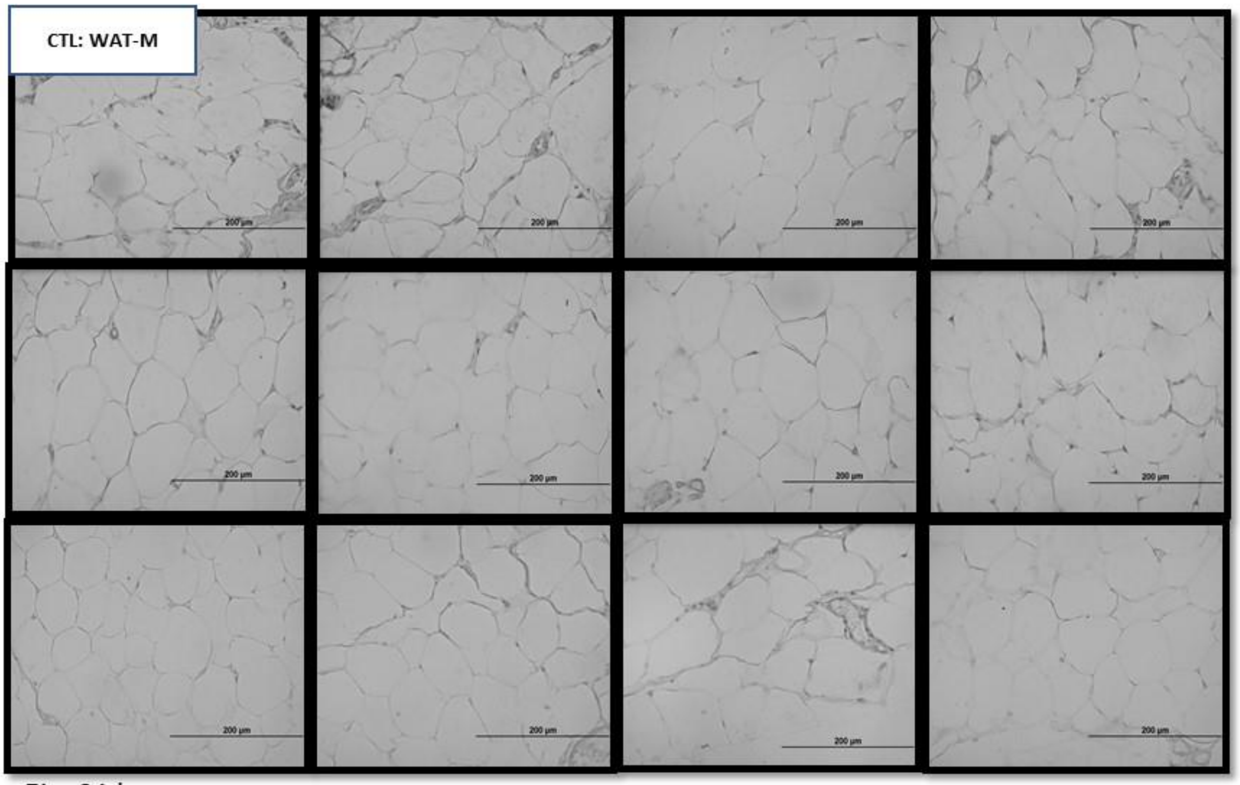

Fig. S1 b

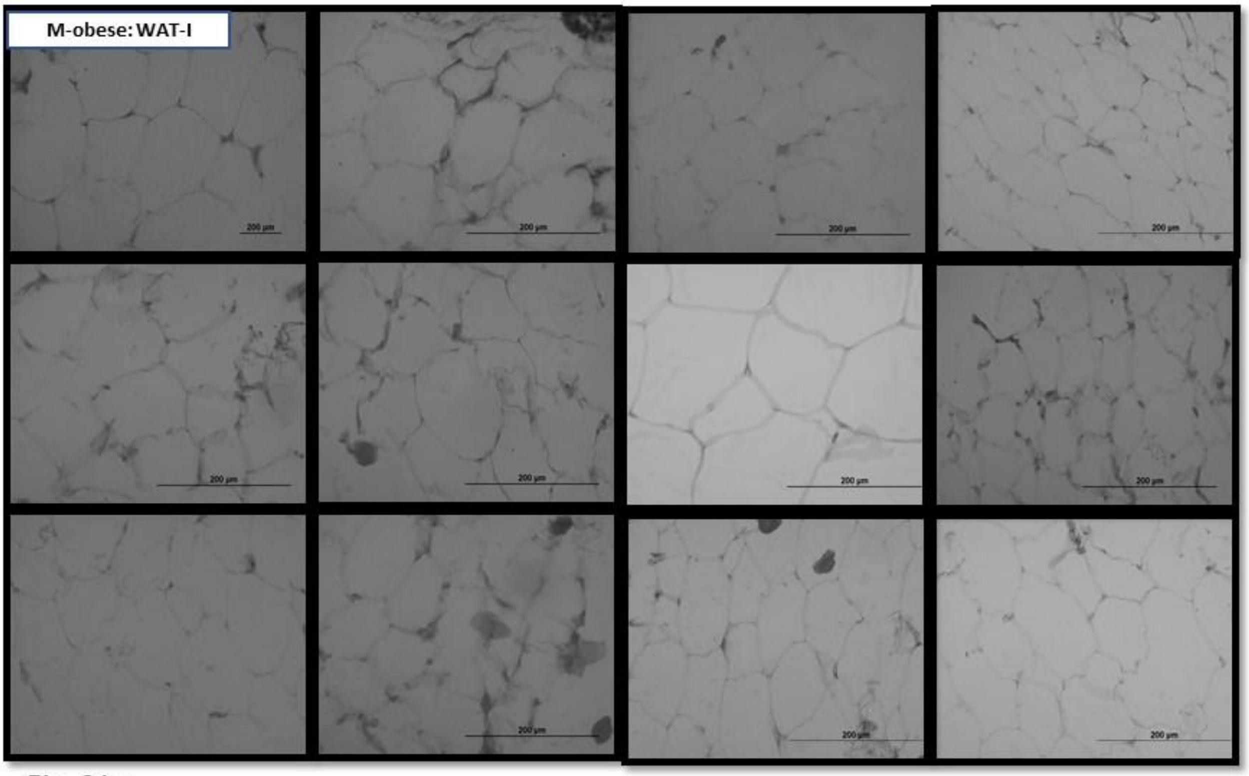

Fig. S1 c

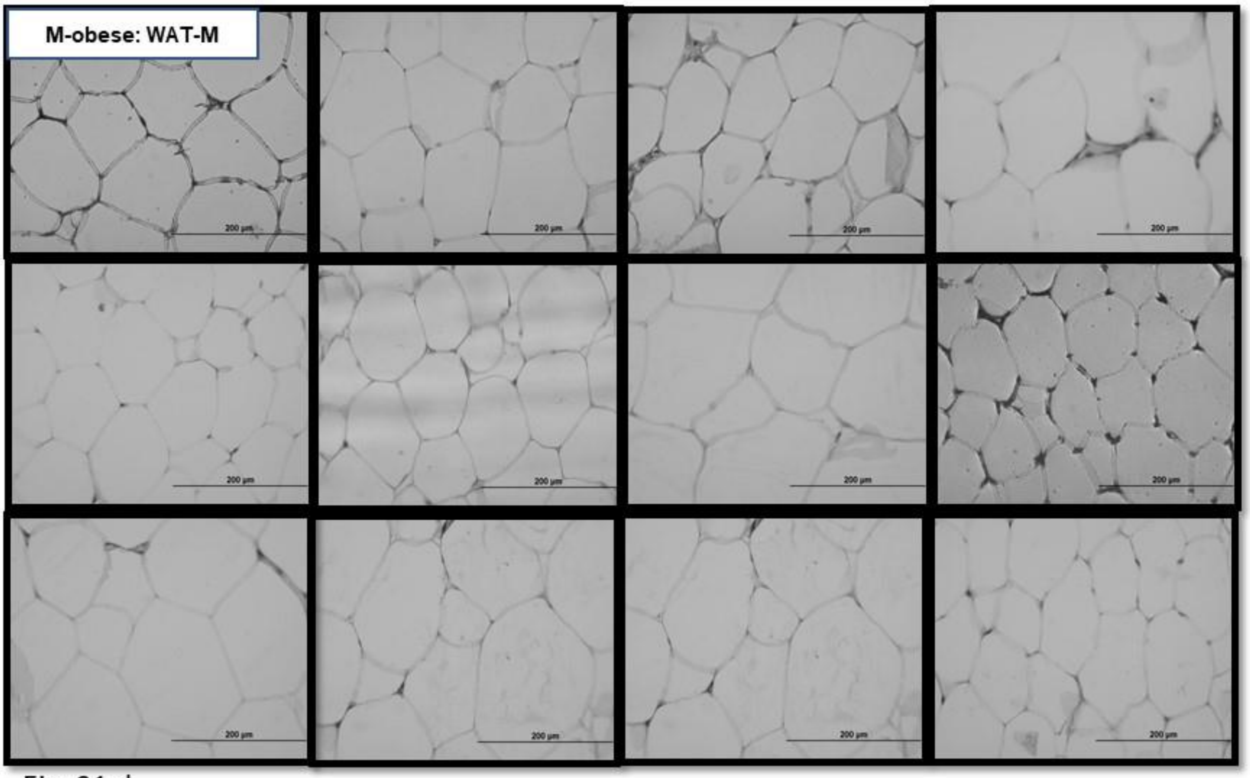

Fig. S1 d

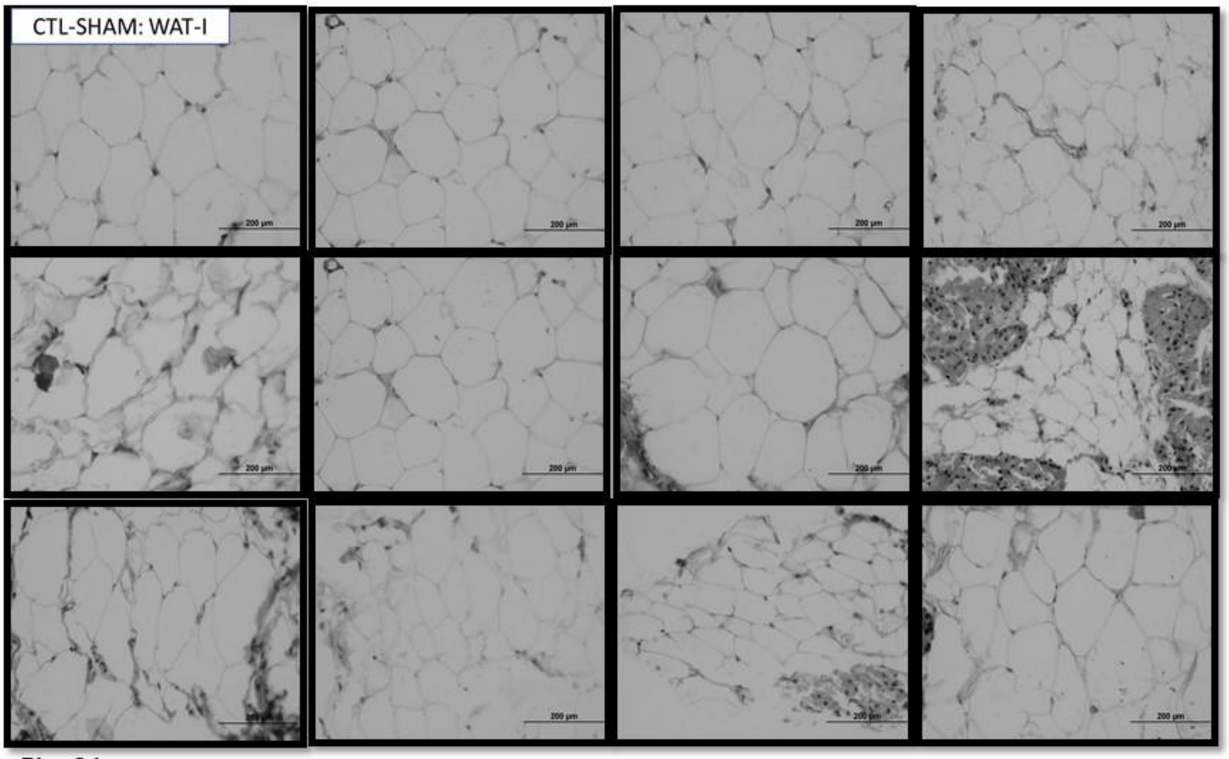

Fig. S1 e

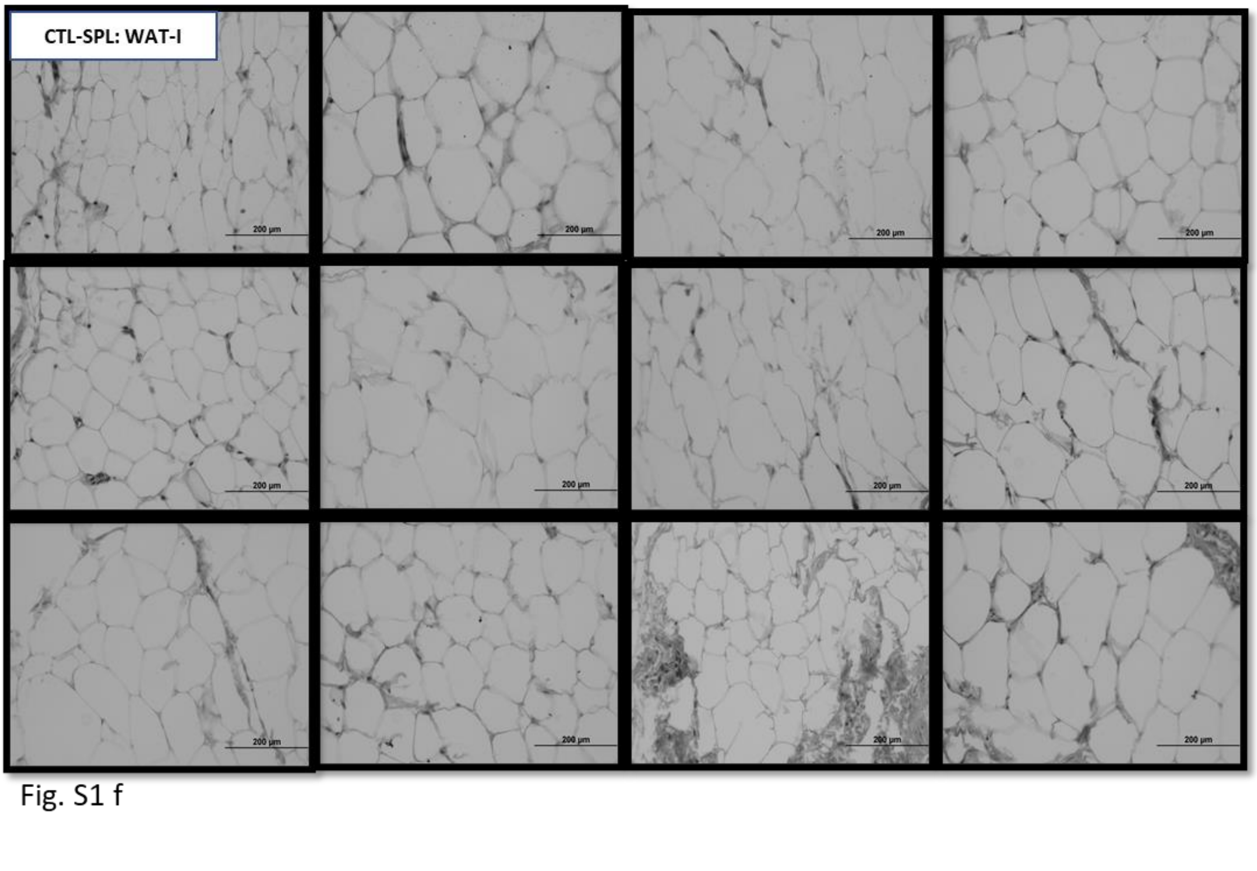

Fig. S1 f

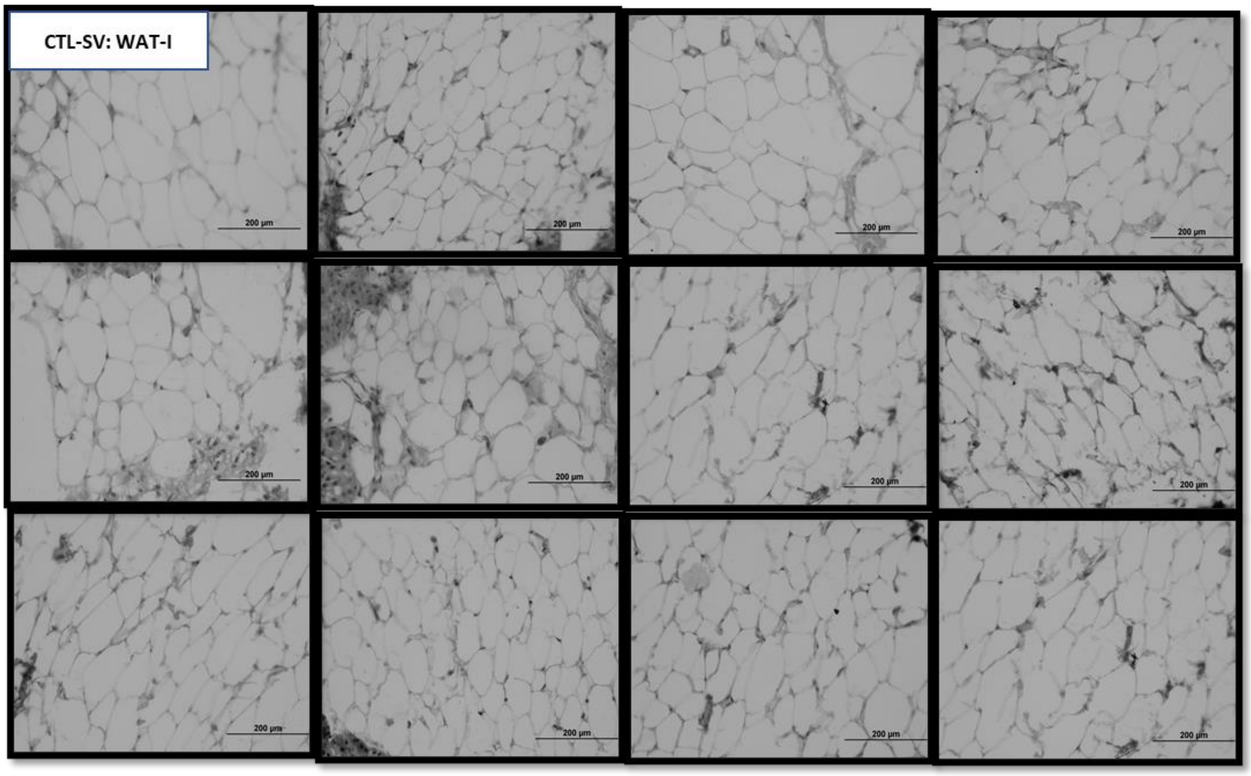

Fig. S1 g

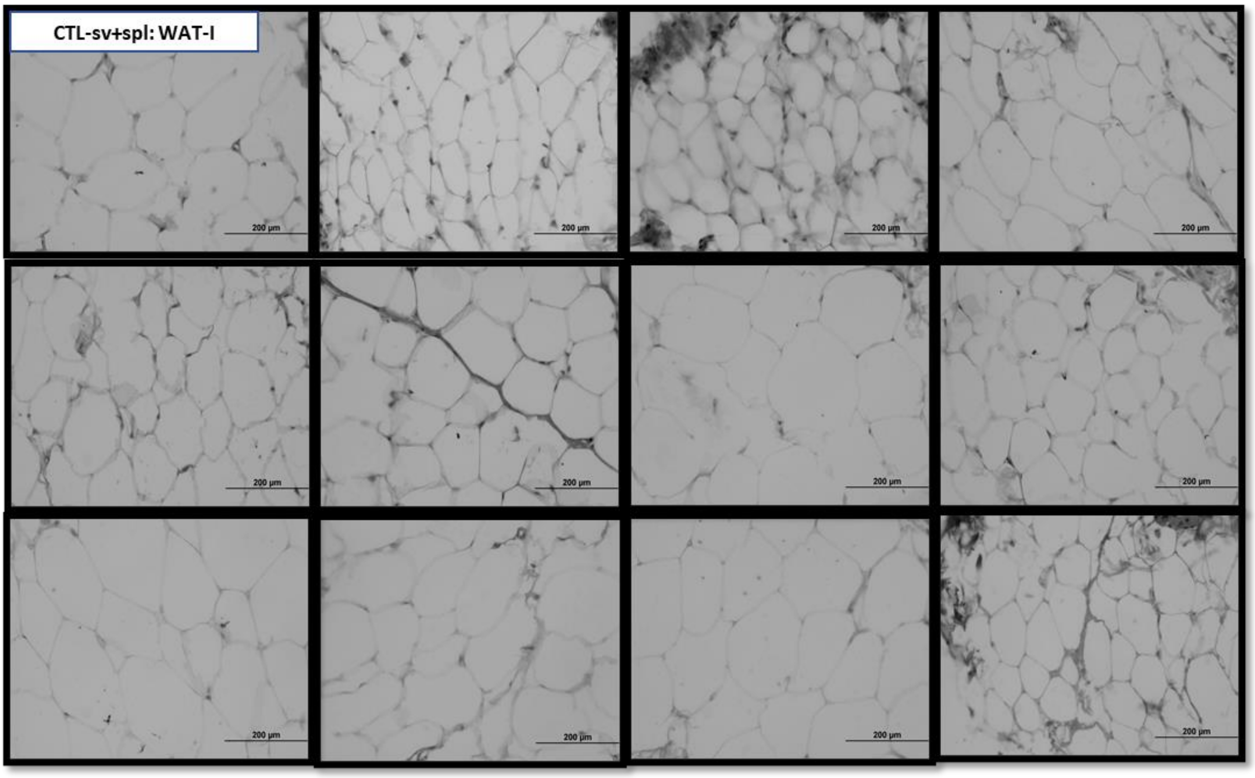

Fig. S1 h

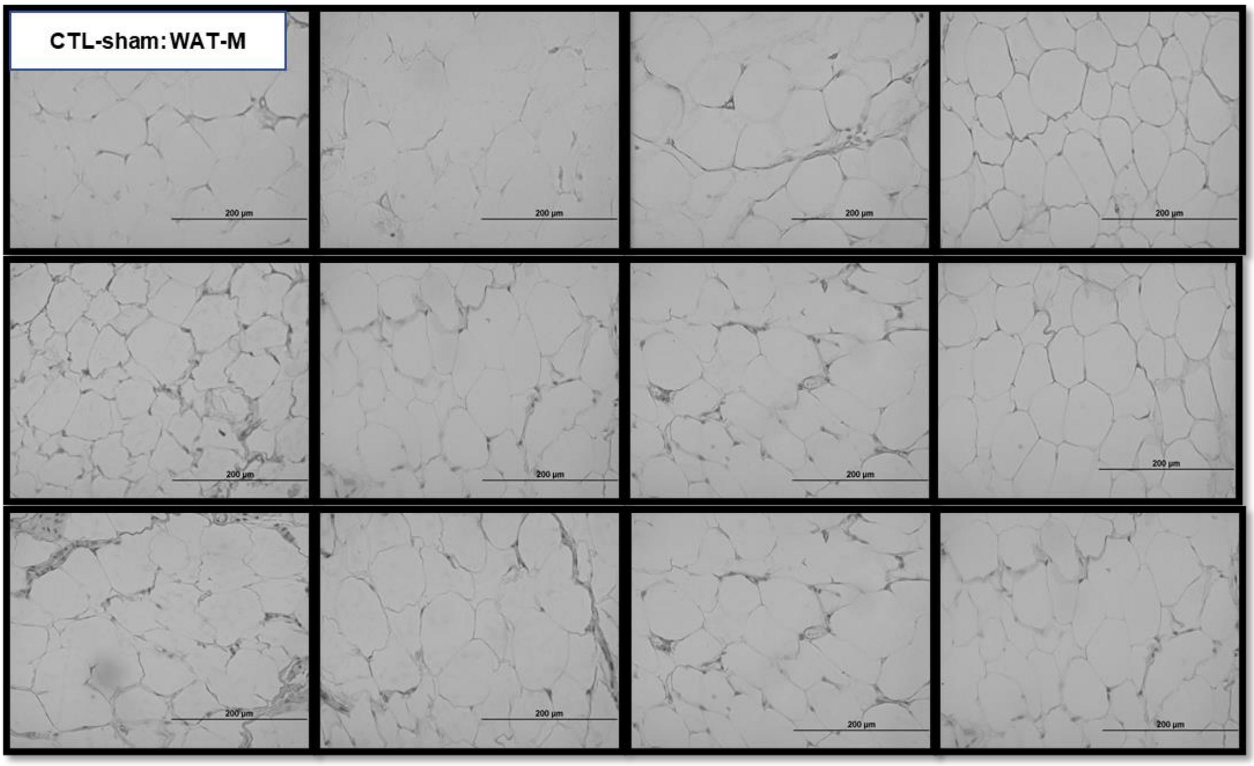

Fig. S1 i

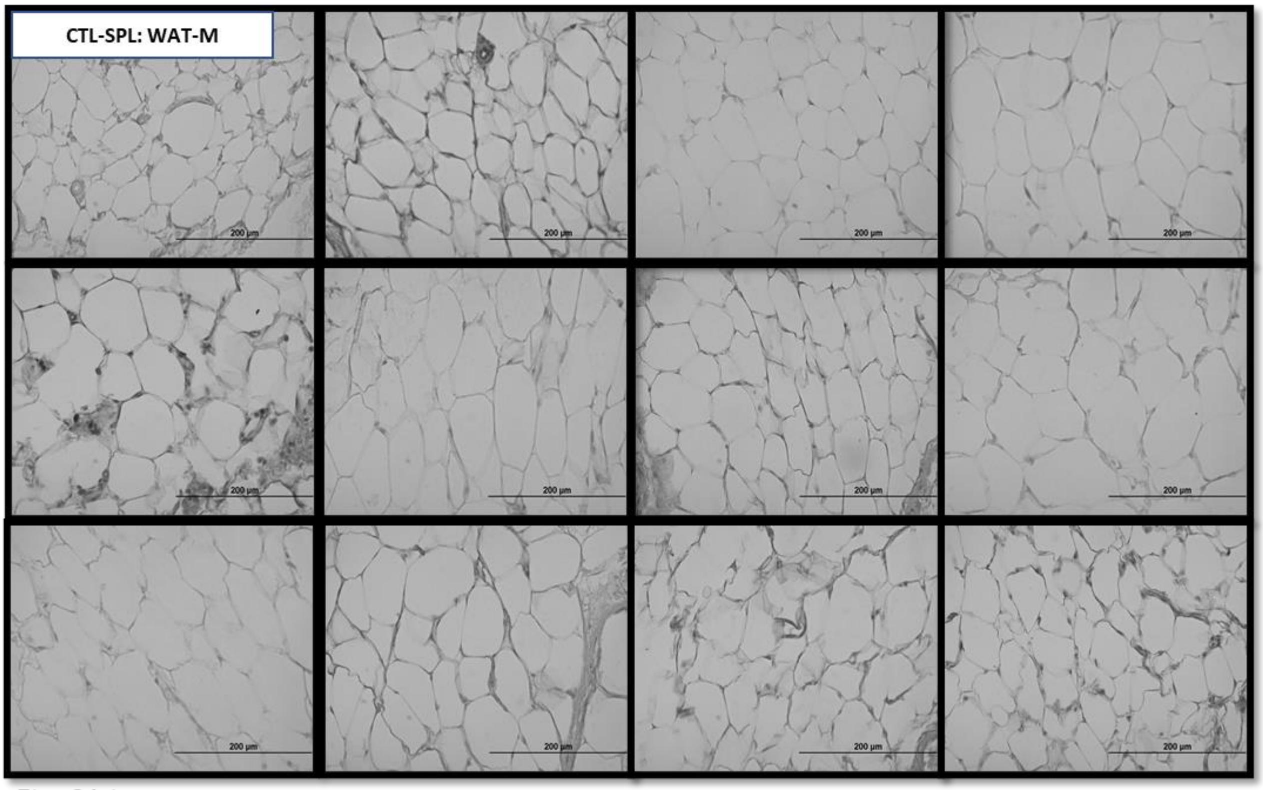

Fig. S1 j

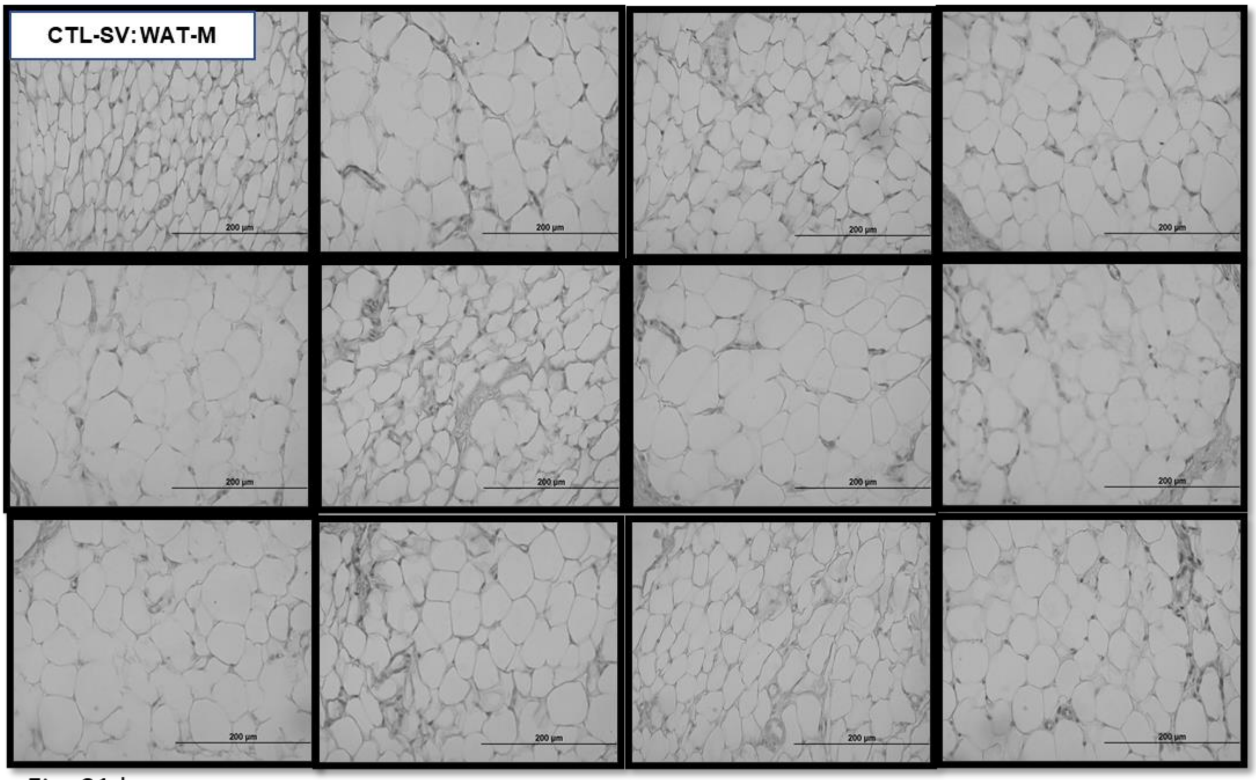

Fig. S1 k

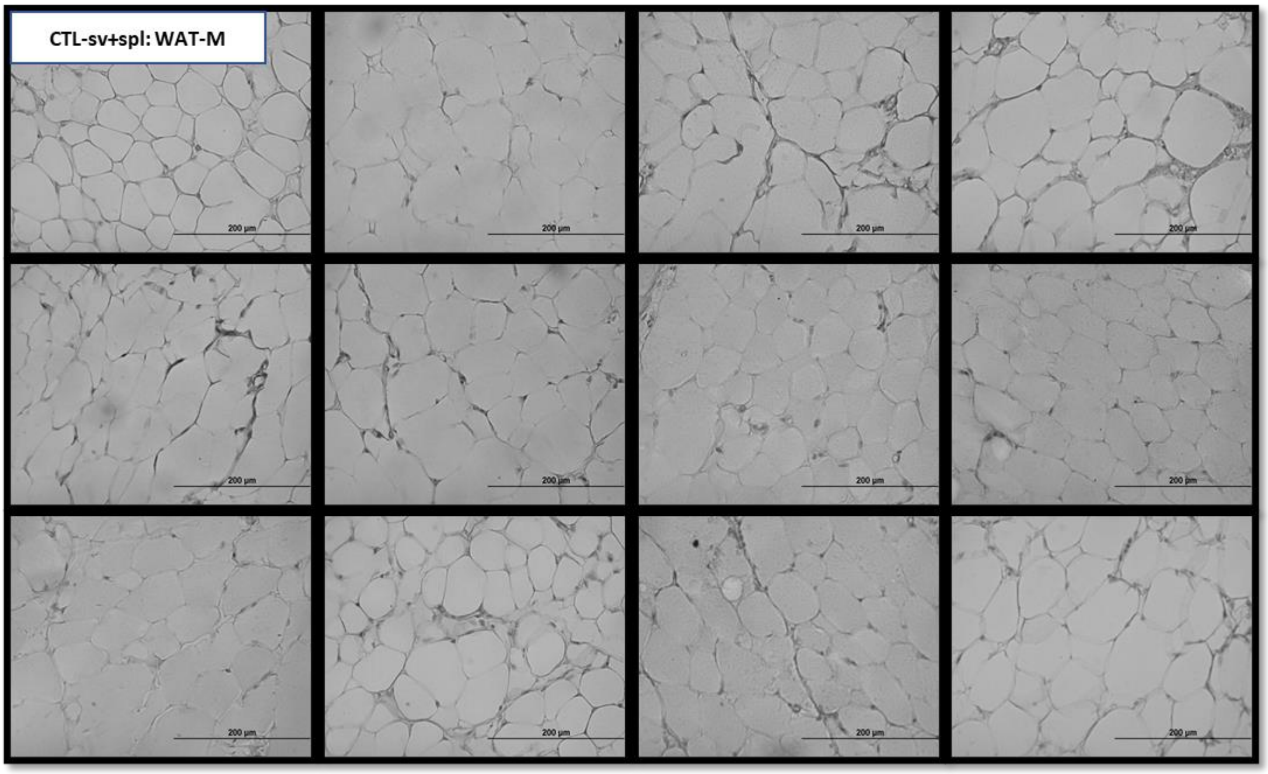

Fig. S1 l

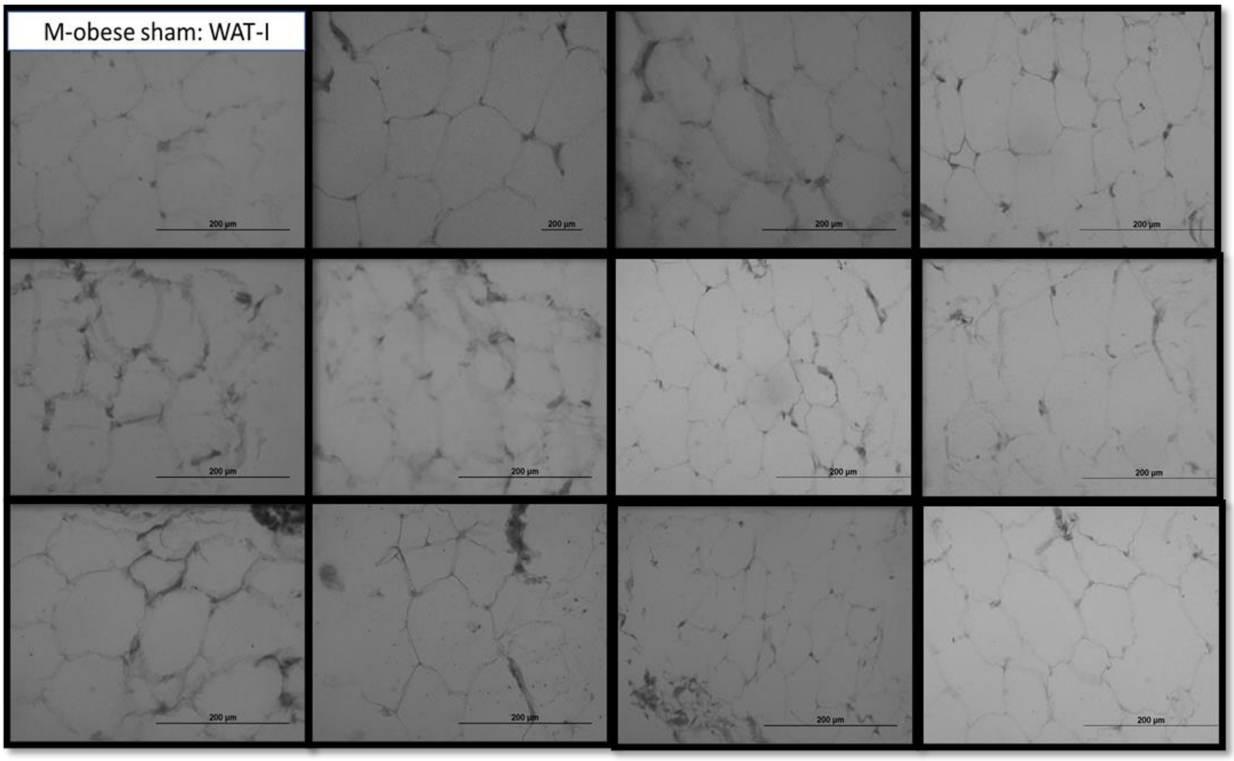

Fig. S1 m

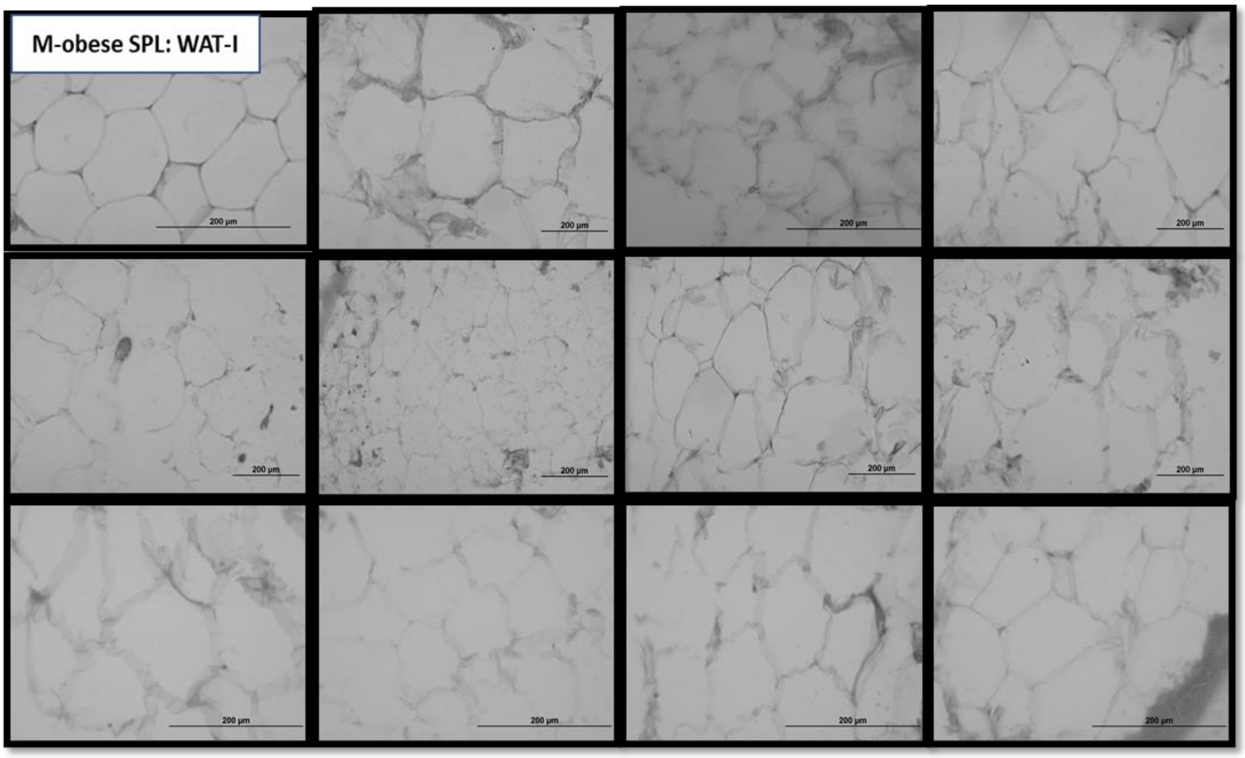

Fig. S1 n

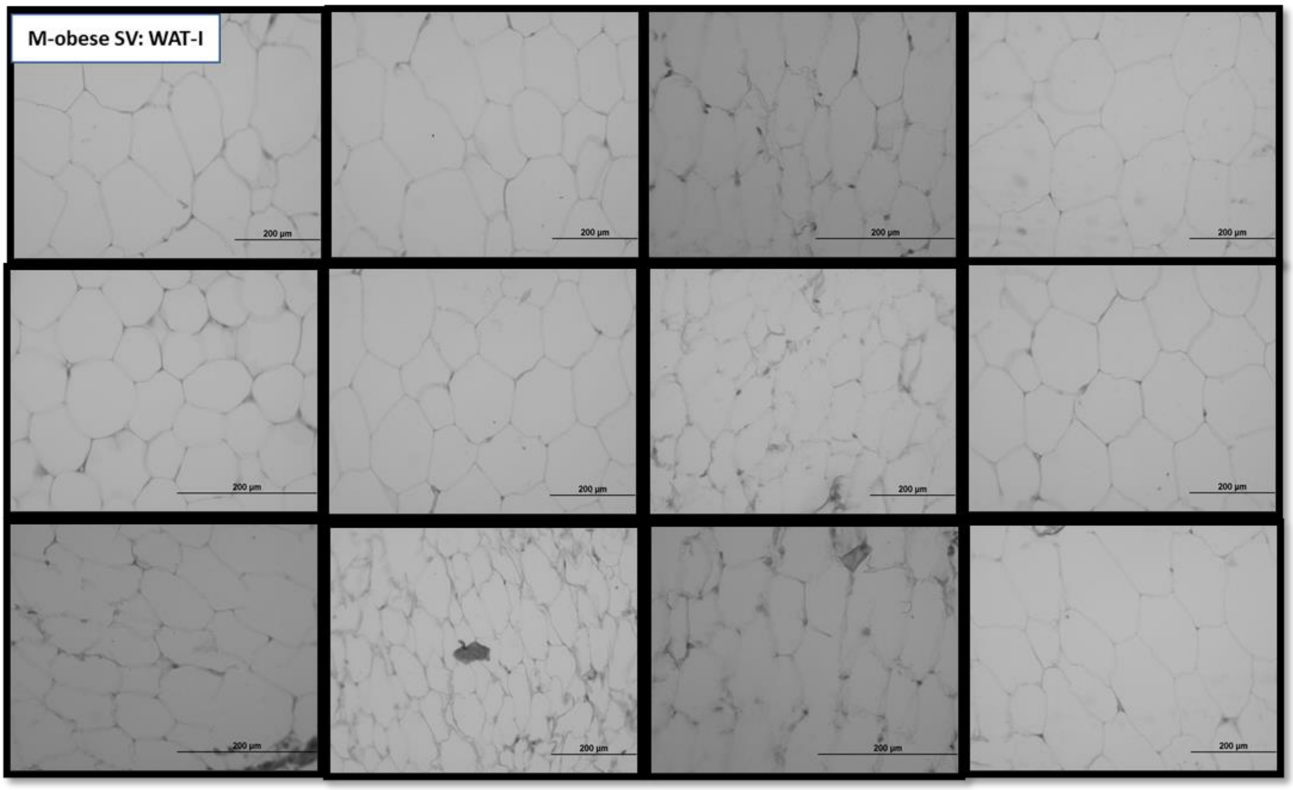

Fig. S1 o

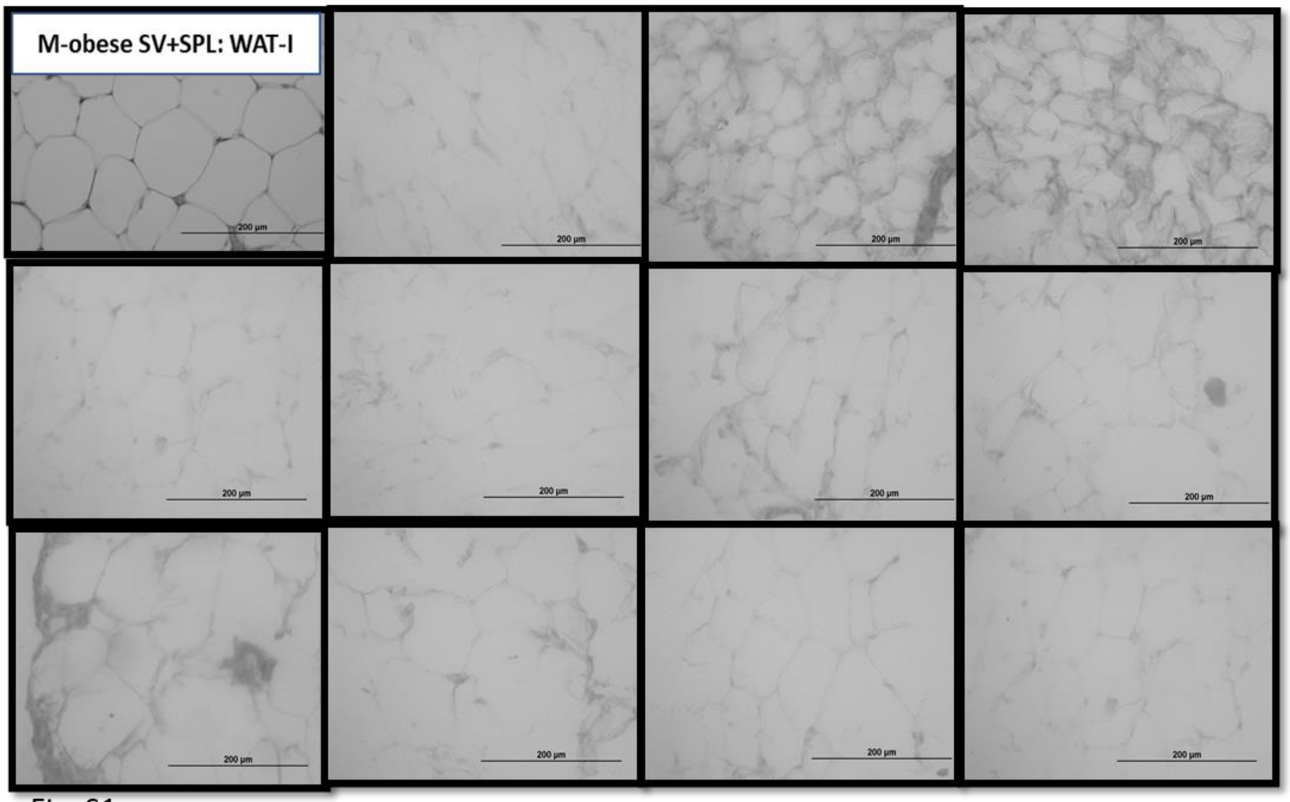

Fig. S1 p

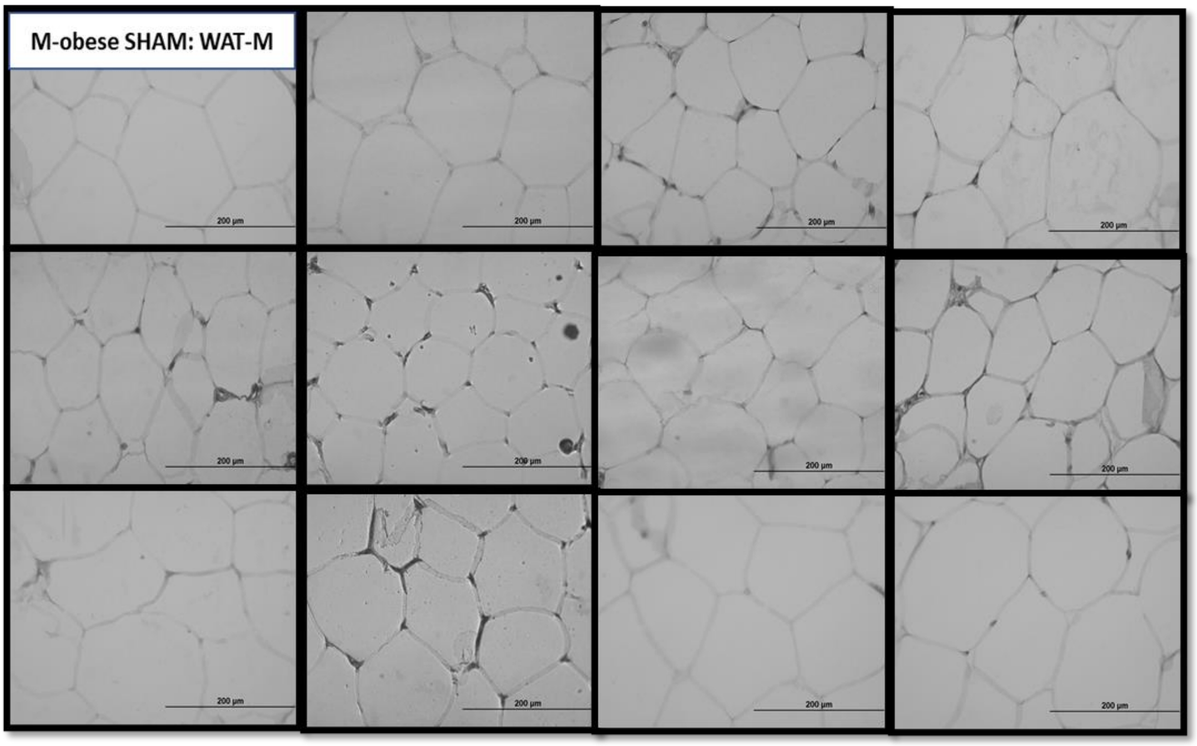

Fig. S1 q

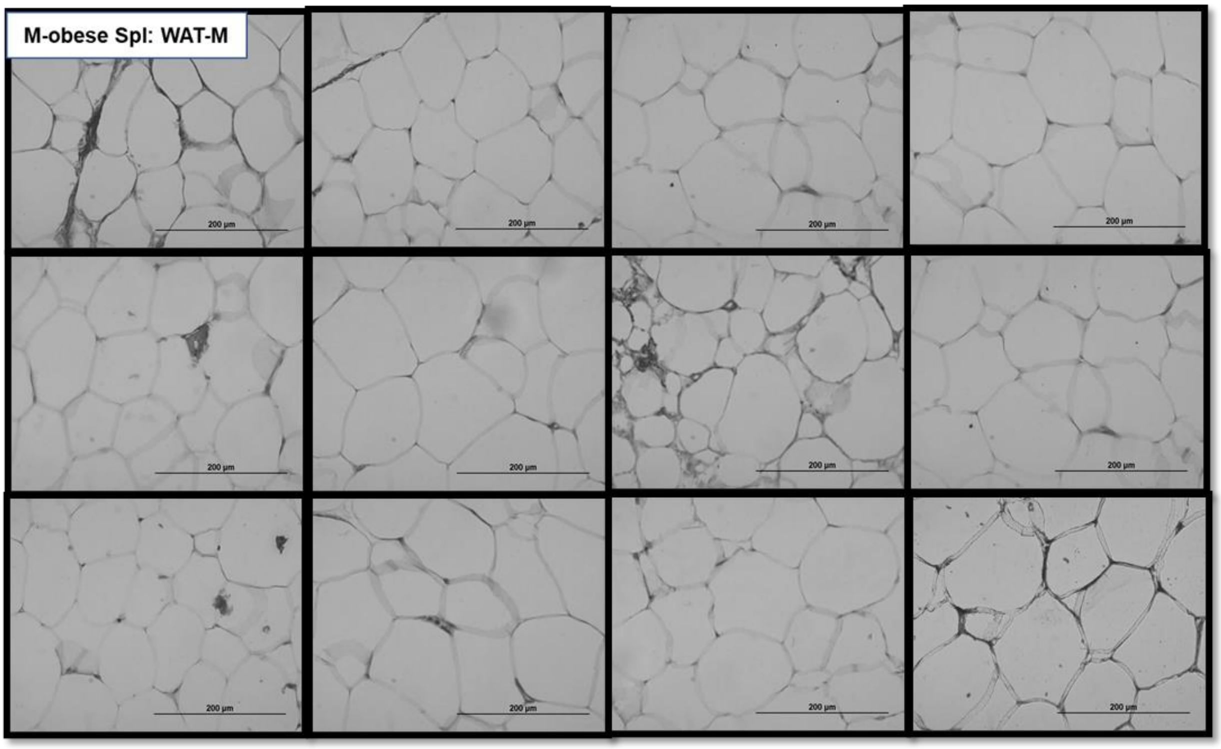

Fig. S1 r

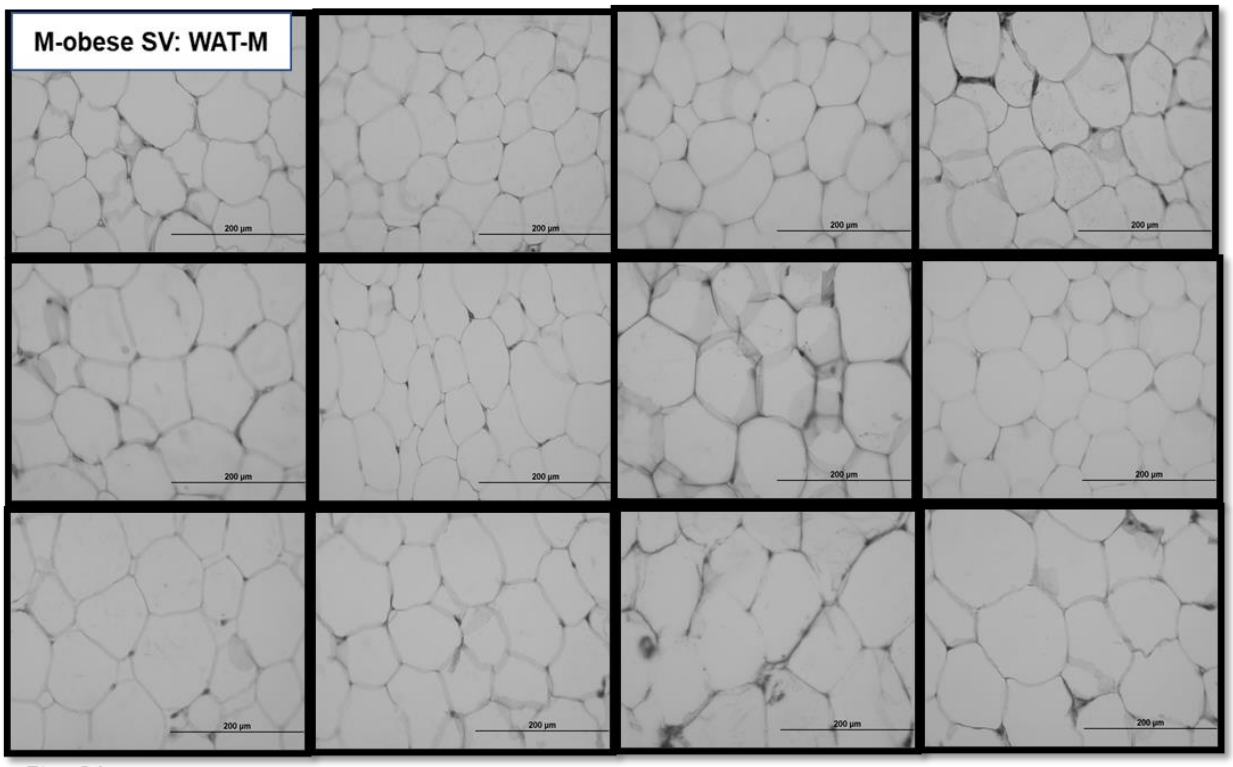

Fig. S1 s

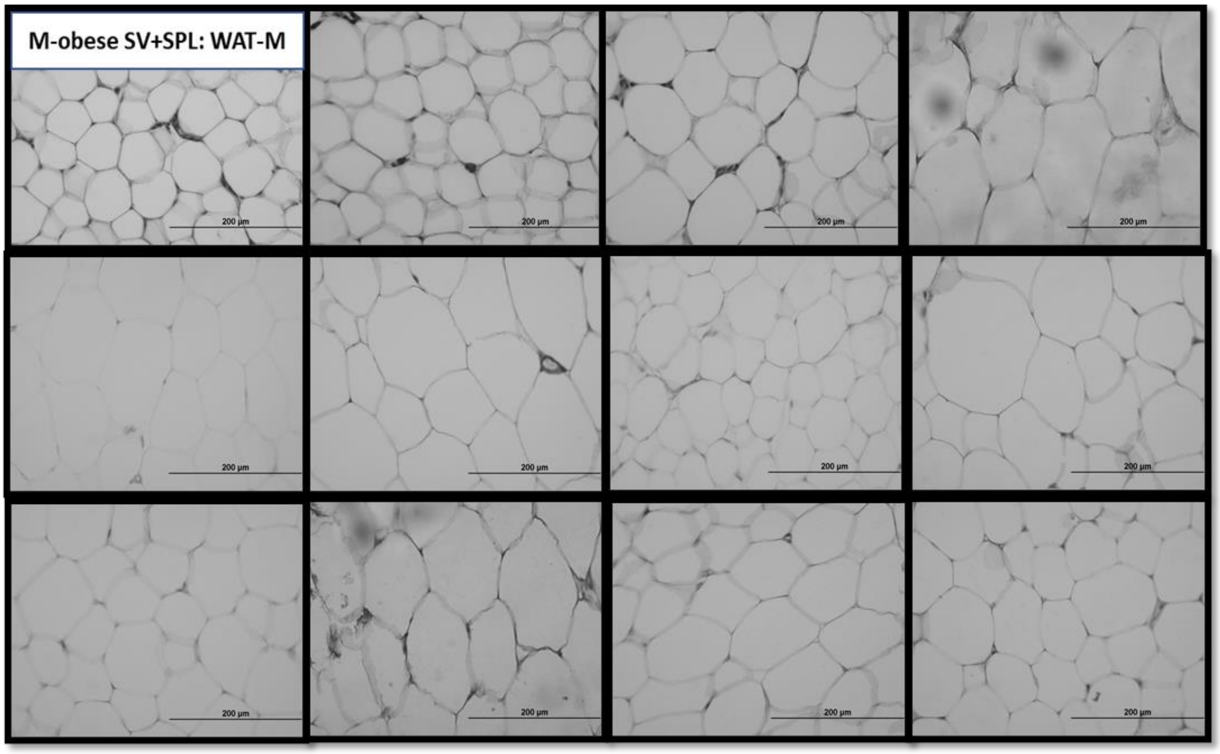

Fig. S1 t

The photomicrographs were stained with H&E, as described in method section and are shown in gray scale; magnification 40x. Eight to twelve lamine were selected from n 4 – 5 rats for group. Legend: WAT: White adipose tissue, I: inguinal; M: mensenteric: CTL: Control; M: monosodium glutamate; H: hematoxylin, E: eosin. CTL (Fig. a and b); M-Obese (Fig. c and d); WAT-I: CTL-sham (Fig. e); CTL-spl (Fig. f); CTL-sv (Fig. g); CTL-sv+spl (Fig. h); WAT-M: CTL-sham (Fig. i); CTL-spl (Fig. j); CTL-sv (Fig. k); CTL-sv+spl (Fig. l); WAT-I: M-Obese-sham (Fig. m); M-Obese-spl (Fig. n); M-Obese-sv (Fig. o); M-Obese-sv+spl (Fig. p); WAT-M: M-Obese-sham (Fig. q); M-Obese-spl (Fig. r); M-Obese-sv (Fig. s); M-Obese-sv+spl (Fig. t);
