# Supplementary material for: The Vagus Nerve and Spleen: Influence on White Adipose Mass and Histology of Obese and Non-obese Rats
Source: Front Physiol. 2021 Jun 25;12:672027. doi: 10.3389/fphys.2021.672027 (PMC8269450; doi:10.3389/fphys.2021.672027)
Supplement: Supplementary file 2 [file Table_1.pdf]

Supplementary Table S1: ***Glass´s delta*** **ES in CTL (non-obese) and M-Obese rats.**

**Variables**	**CTL**			**M-Obese**		
	**SPL vs SHAM**	**SV vs SHAM**	**SV+SPL vs SHAM**	**SPL vs SHAM**	**SV vs SHAM**	**SV+SPL vs SHAM**
**Biometric**	**ES**	**ES**	**ES**	**ES**	**ES**	**ES**
Body weight gain	-1.24	-3.25	-3.23	-0.54	-1.57	-1.15
NAL	-0.38	-2.41	-1.67	0.64	1.00	0.61
Lee index	-0.91	0.28	-0.85	-1.39	-2.0	-2.40
WAT-I	0.95	-0.80	-0.49	0.28	0.67	-0.00
WAT-M	0.02	-2.58	-1.47	0.25	-1.21	-1.24
Stomach	0.93	12.37	11.76	-0.60	4.67	7.24
Spleen	N/A	-0.46	N/A	N/A	-0.24	N/A
**Biochemical**						
Glycemia	-0.82	0.41	2.60	0.31	0.53	0.74
Insulin	0.13	1.74	1.72	0.40	-0.73	0.68
Cholesterol	8.69	3.36	6.50	-0.34	-0.70	-0.47
Triglycerides	0.24	-0.88	-1.17	0.55	-2.02	-1.88
TyG index	-0.69	-0.95	0.084	-0.84	-2.17	-2.01
IL-10 plasma	0.69	3.13	-0.58	0.30	0.61	0.60
IL-10 WAT-I	-0.15	-0.70	-0.20	0.94	1.82	1.28
IL-10 WAT-M	-0.03	-0.50	0.24	-0.15	-0.76	-0.28
**Histological**						
Adipocytes size WAT-I	0.22	-1.09	0.50	-1.27	-2.88	-2.33
Adipocytes number WAT-I	0.35	3.21	0.33	-0.45	3.96	2.11
Adipocytes size WAT-M	-1.61	-4.14	-2.06	-0.82	-1.17	-1.44
Adipocytes number WAT-M	0.65	4.74	2.32	0.67	2.83	3.50

Glass´s delta values (effect size) were defined as the difference among the means, divided by SD of CTL. Relevance was interpreted as small (delta = 0.2), medium (delta = 0.5), or large (delta = 0.8). Legend: SHAM; surgical simulation; SPL, splenectomy; SV, subdiaphragmatic vagotomy; M, Monosodium glutamate; ES, effect size; NAL, Naso-anal length; WAT-I, Inguinal white adipose tissue; WAT-M, Mesenteric white adipose tissue; IL10, Interleukin 10; N/A, not applicable.
